# The CXCL10‐CXCR3 Axis Induces Tumor‐Associated Neutrophils to Interfere with CTLs‐Mediated Antitumor Activity in EBV‐Associated Epithelial Cancers

**DOI:** 10.1002/advs.202500950

**Published:** 2025-07-21

**Authors:** Dijun Ouyang, Tong Xiang, Yuanyuan Chen, Mengjia Song, Jingjing Zhao, Hao Chen, Si Li, Lifeng Zhang, Chi Xu, Yan Ren, Yong Tao, Qijing Wang, Jia He, Yongqiang Li, Sisi Xie, Yuanyuan Liu, Yan Wang, Xinyi Yang, Jinqi You, Songzuo Xie, Yingzi Li, Desheng Weng, Qiuzhong Pan, Qi Yang, Jianchuan Xia

**Affiliations:** ^1^ State Key Laboratory of Oncology in South China Guangdong Provincial Clinical Research Center for Cancer Guangdong Key Laboratory of Nasopharyngeal Carcinoma Diagnosis and Therapy Sun Yat‐sen University Cancer Center Guangzhou Guangdong 510060 P. R. China; ^2^ Department of Biotherapy Sun Yat‐sen University Cancer Center Guangzhou Guangdong 510060 P. R. China; ^3^ Department of Experimental Research Sun Yat‐sen University Cancer Center Guangzhou Guangdong 510060 P. R. China; ^4^ Department of Pediatric Oncology Sun Yat‐sen University Cancer Center Guangzhou Guangdong 510060 P. R. China; ^5^ TCRCure Biological Technology Co., Ltd Guangzhou China; ^6^ Knowcell Biotechnology Co., Ltd Shenzhen China; ^7^ Department of Medical Oncology The Third Affiliated Hospital of Sun Yat‐sen University 600 Tianhe Road Guangzhou 510630 China; ^8^ Department of Nuclear Medicine Sun Yat‐sen University Cancer Center Guangzhou 510060 P. R. China; ^9^ Department of Nasopharyngeal Carcinoma Sun Yat‐sen University Cancer Center Guangzhou 510060 P. R. China

**Keywords:** EBV, EBVaGC, NPC, neutrophil extracellular traps, tumor‐associated neutrophils

## Abstract

Although Epstein–Barr virus (EBV)‐associated epithelial cancers are categorized as immunologically “hot” tumors, they have unsatisfactory responses to immunotherapy. Increasing evidence has shown that therapeutic failure is due to an immunosuppressive tumor microenvironment established by EBV. In this study, a negative correlation is found between the infiltration of neutrophils and that of cytotoxic T lymphocytes (CTLs) containing granzyme B in EBV‐associated epithelial cancers. The CXCL10‐CXCR3 axis in EBV‐associated epithelial cancer cells controls the extrusion of neutrophil extracellular traps (NETs), which interferes with the antitumor activity of EBV antigen‐specific T cells in vitro and in vivo. NETs are positively correlated with the number of dysfunctional CTLs in EBV‐associated epithelial cancers, and are confirmed to be an independent prognostic factor for patients with EBV‐associated epithelial cancers. In conclusion, these findings reveal a novel mechanism of immunosuppression of tumor‐associated neutrophils (TANs) in EBV‐associated epithelial cancers. Targeting NETs formation in TANs may be a potential strategy for improving the efficacy of immunotherapy against EBV‐associated epithelial cancers.

## Graphical abstract

Compared with EBV‐negative epithelial cancer cells, EBV‐associated epithelial cancer cells exhibit enhanced secretion of chemokines, including CXCL10 and CCL5, to attract neutrophils. Via the CXCL10‐CXCR3 axis‐mediated positive feedback loop, neutrophils within EBV‐associated epithelial cancer cells are induced to form abundant NETs, which facilitate the growth of tumor cells by interfering with CTLs‐mediated cytotoxicity.

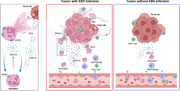

## 1 Introduction

Epstein–Barr virus (EBV), a member of the Herpesviridae family, is one of the most common human viruses, affecting more than 90% of the global population.^[^ ^1^ ^]^ While EBV is typically associated with benign conditions or does not cause major symptoms in the majority of lifelong carriers, it has also been implicated in the development of a range of lymphoma, such as Burkitt lymphoma, classical Hodgkin lymphoma and NK/T‐cell lymphoma, and epithelial malignancies.^[^ ^2^ ^]^ Nasopharyngeal carcinoma (NPC) and EBV‐associated gastric carcinoma (EBVaGC) are the most common EBV‐associated epithelial cancers, accounting for 80% of all EBV‐associated malignancies.^[^ ^3^ ^]^ NPC is one of the most common EBV‐associated epithelial cancers in Southeast Asia and parts of North Africa. Approximately 95% of NPC cases are EBV‐positive non‐keratinizing NPC,^[^ ^4^ ^]^ whereas EBVaGC accounts for ≈10% of all gastric cancers.^[^ ^5^ ^]^

NPC and EBVaGC with an abundance of CD8^+^ T cells are known as “hot” tumors.^[^ ^5^, ^6^ ^]^ However, exhausted T cells are also found in these “hot” tumors,^[^ ^7^ ^]^ leading to tumor immune escape. Immunotherapy, including immune checkpoint inhibitors (ICIs) and adoptive cell therapy (ACT), has emerged as a pivotal treatment to overcome the mechanisms of tumor immune escape.^[^ ^8^ ^]^ Clinical trials have shown that ICIs do not achieve the expected efficacy in patients with NPC and EBVaGC.^[^ ^9^, ^10^, ^11^ ^]^ In addition, ACT has shown limited benefit for patients with NPC in clinical trials.^[^ ^12^, ^13^ ^]^ These phenomena indicate immunosuppression in EBV‐associated epithelial cancers.

An increasing number of studies have focused on elucidating how EBV facilitates the maintenance of immunosuppression. For example, EBV‐driven latent membrane proteins, cytokines and microRNAs reportedly upregulate PD‐L1 expression in NPC and EBVaGC.^[^ ^14^, ^15^, ^16^ ^]^ In addition, immune cells in the tumor microenvironment (TME) play an important role in managing immunosuppression in EBV‐associated epithelial cancers. Treg cells are promoted by Epstein‐Barr nuclear antigen 1^[^ ^17^ ^]^ and EBV‐dependent epigenetic modifications of NPC cells,^[^ ^18^ ^]^ resulting in an immunosuppressive microenvironment. Macrophages polarize into M2 macrophages in NPC via EBV‐induced CSF‐1 and ATR, resulting in tumor progression.^[^ ^7^, ^19^, ^20^ ^]^ However, the TME in EBV‐associated epithelial cancers is a complex and dynamic ecosystem and is not yet completely understood.

Neutrophils in the TME, known as tumor‐associated neutrophils (TANs), have gained increasing attention in recent years. TANs are reported to play a paradoxical role, influencing both tumor progression and suppression.^[^ ^21^ ^]^ TANs play a role through phagocytosis; the release of reactive oxygen species (ROS), granules, and enzymes; or the formation of neutrophil extracellular traps (NETs), which are manipulated by tumors and, in turn, modulate the TME.^[^ ^22^ ^]^ NETs are web‐like structures containing extracellular DNA fibers and associated granule proteins extruded by neutrophils. NETs exert antitumor effects by inducing apoptosis and inhibiting the proliferation or migration of tumor cells^[^ ^23^, ^24^ ^]^ and exert protumor effects by promoting metastasis,^[^ ^25^, ^26^ ^]^ awakening dormant cancer cells^[^ ^27^ ^]^ and shielding cancer cells from cytotoxicity.^[^ ^28^ ^]^ They can also create an immunosuppressive environment by inhibiting the activation and function of other immune cells, such as T lymphocytes.^[^ ^29^, ^30^ ^]^ NETs are formed by a process termed NETosis. Classically, NETosis depends on ROS production and peptidyl arginine deiminase 4 (PAD4). PAD4 is a Ca2^+^‐specific enzyme primarily localized in the nucleus that modifies histones by changing arginine to citrulline, leading to chromatin decondensation, and upon nuclear rupture, citrullinated histones together with nuclear DNA are released.^[^ ^31^ ^]^ This process is induced by various factors, such as chronic stress,^[^ ^32^ ^]^ cathepsin C,^[^ ^25^ ^]^ and chemokines.^[^ ^28^ ^]^

In this study, we have investigated how EBV‐associated epithelial cancer cells induce NETs formation and how NETs affect the cytotoxicity of killing cells to promote the progression of EBV‐associated epithelial cancers. Understanding these mechanisms could lead to the development of novel therapeutic strategies aimed at reprogramming NETs or TANs to mitigate their protumor effects, thereby improving the efficacy of immunotherapy and leading to better clinical outcomes for patients with EBV‐associated epithelial cancers.

## 2 Results

### 2.1 TANs in EBV‐Associated Epithelial Cancers Are Negatively Correlated with the Number of CD8^+^ T Cells

**Figure 1:**
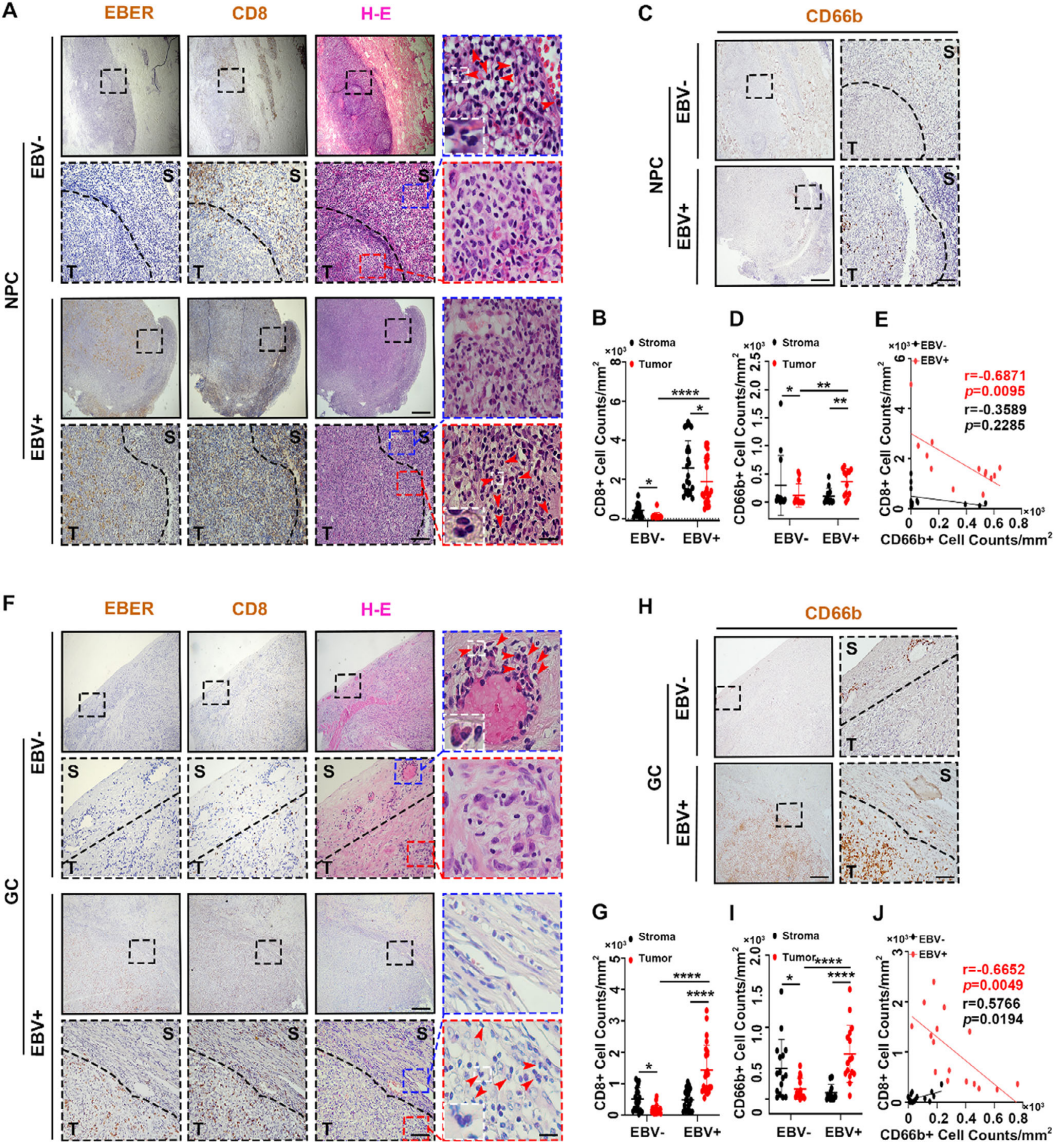
TANs in EBV‐associated epithelial cancers are negatively correlated with the number of CD8^+^ T cells. A,F) Representative images of EBV^‐^ and EBV^+^ NPC (A) and GC (F) sections stained for EBER, CD8 and H‐E. Magnifications: 40x (black solid line frame), 200x (black dashed line frame), 1000x (blue and red dashed line frame). “S” indicates the stroma area, presented in the blue dashed line frame; “T” indicates the tumor area, presented in the red dashed line frame. The white dashed line frame and red arrows represent neutrophils. Scale bars: 500 µm (40x images), 100 µm (200x images), 20 µm (1000x images). B,G) Statistical analysis of the density of CD8^+^ cells in NPC (B) and GC (G). C,H) Representative images of serial sections from EBV^‐^ and EBV^+^ NPC (C) and GC (H) stained for CD66b. Magnifications: 40x (black solid line frame), 200x (black dashed line frame). “S” indicates the stroma area, and “T” indicates the tumor area. Scale bars: 500 µm (40x images), 100 µm (200x images). D,I) Statistical analysis of the density of CD66b^+^ cells in NPC (D) and GC (I). E,J) Correlation analysis between CD66b^+^ cells and CD8^+^ cells in NPC (E) and GC (J). NPC: nasopharyngeal cancer. GC: gastric cancer. EBER: Epstein–Barr virus‐encoded small RNA. Mean ± SD are shown for all panels, including error bars. *p*‐values were calculated with Mann–Whitney *U* test or two‐tailed *t*‐test. ^*^ *p* < 0.05, ^**^ *p* < 0.01, ^***^ *p* < 0.001, and ^****^ *p* < 0.0001, n.s., not significant.

EBV‐associated epithelial cancers are immunologically “hot,” with a high abundance of cytotoxic T lymphocytes (CTLs). These CTLs are reported to be exhausted or ineffective. To uncover the mechanism underlying this phenomenon, we first utilized EBV‐associated cancer samples from patients with NPC or GC to detect the infiltration of CD8^+^ T cells. As expected, more CD8^+^ T cells infiltrated the tumor area of EBV‐positive epithelial cancers comparing with EBV‐negative epithelial cancers (**Figure** **1**A,B,F,G). Focusing on these CD8^+^ T cells, we observed that they were surrounded by classically morphological neutrophils, characterized by their lobulated and hyper‐segmented‐shaped nuclei^[^ ^21^ ^]^ (Figure 1A,F). To further confirm this, we performed conventional immunohistochemical staining for the neutrophil marker, CD66b.^[^ ^33^ ^]^ We observed that CD66b^+^ cells were present mainly in the tumor area of EBV‐positive epithelial cancers and in the stroma of EBV‐negative epithelial cancers (Figure 1C,D,H,I). More importantly, a negative correlation was observed between CD8 expression and CD66b expression in the tumor area of EBV‐positive epithelial cancers, while not in the tumor area of EBV‐negative epithelial cancers (Figure 1E,J). Due to its association with neutrophil activation, myeloperoxidase (MPO) serves as an important neutrophil marker in many research.^[^ ^34^ ^]^ To further explore the relationship between neutrophils and CD8^+^ T cells, we also analyzed the results of MPO staining. It was showed that in the tumor area of EBV‐positive epithelial cancers, the number of MPO^+^ cells was higher than that in the tumor area of EBV‐negative epithelial cancers (Figure S1A,B,D,E, Supporting Information). This number was consistently negatively correlated with the number of CD8^+^ T cells (Figure S1C,F, Supporting Information). Overall, these data indicated that the infiltration of CD8^+^ T cells was negatively associated with the infiltration of activated tumor‐associated neutrophils (TANs) in EBV‐associated epithelial cancers.

### 2.2 TANs in EBV‐Associated Epithelial Cancers Inhibit the Cytotoxicity of CTLs

**Figure 2:**
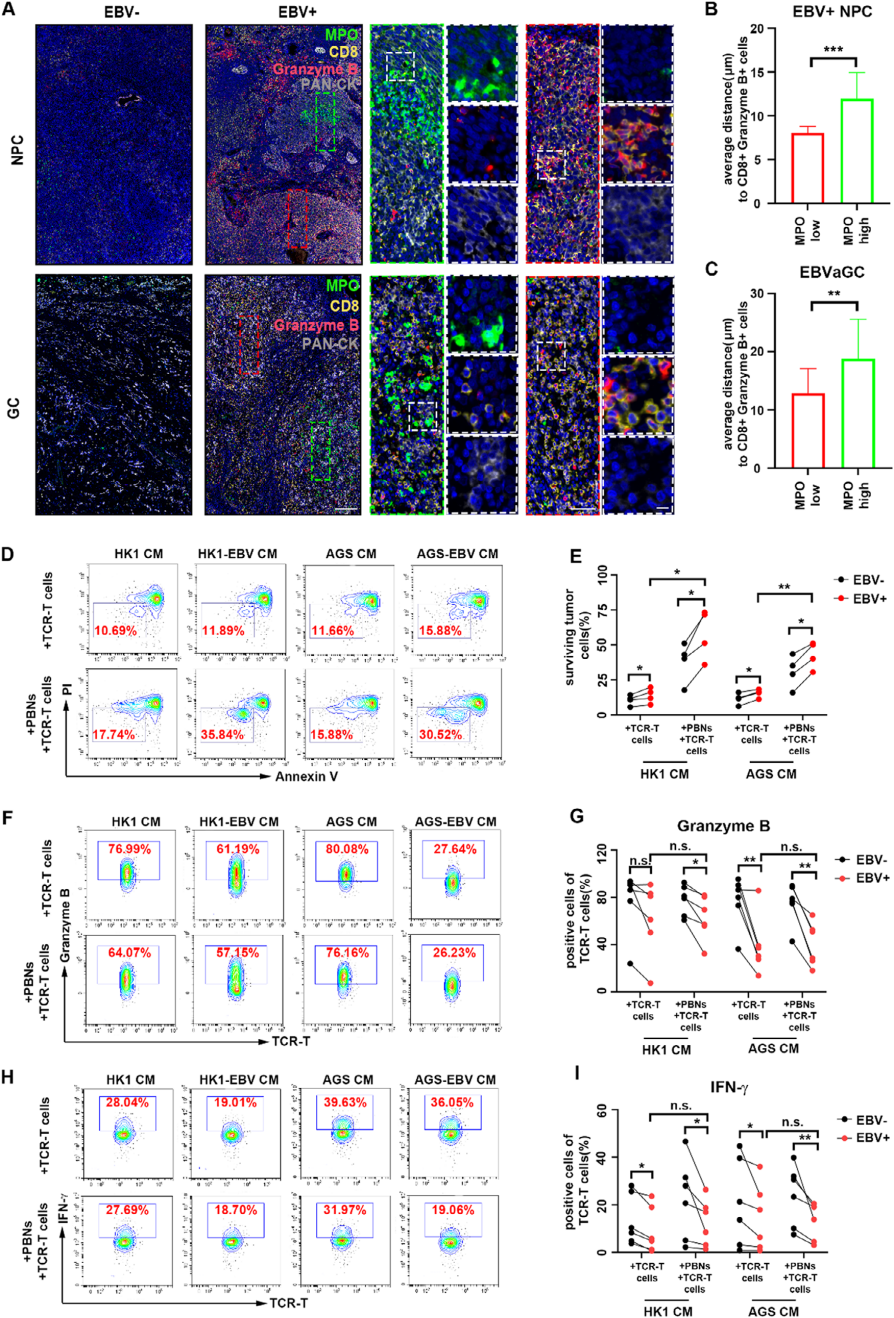
TANs in EBV‐associated epithelial cancers inhibit the cytotoxicity of CTLs. A) Representative images of NPC (upper panel) and GC (lower panel) sections subjected to multiplex immunohistochemistry staining for MPO (green), CD8 (yellow), Granzyme B (red), PAN‐CK (gray), and DAPI. The green dashed line frames represent the area with high MPO expression but low CD8 expression and low granzyme B expression in EBV^+^ NPC (upper panel) and EBVaGC (lower panel); the red dashed line frames represent the area with low MPO expression but high CD8 expression and high granzyme B expression in EBV^+^ NPC (upper panel) and EBVaGC (lower panel); the white dashed line frames represent the single‐stained images of MPO (green) and PAN‐CK (gray), and the merged images of CD8 (yellow) and Granzyme B (red). Magnifications: 40x (black solid line frame), 200x (green and red dashed line frame), 1000x (white dashed line frame). Scale bars: 200 µm (40x images), 50 µm (200x images), 10 µm (1000x images). B,C) Nearest neighbor analysis was conducted to analyze the distance between tumor cells and the nearest CD8^+^ granzyme B^+^ cells in regions with high or low expression of MPO in EBV^+^ NPC (B) and EBVaGC (C), as determined by multiplex immunohistochemistry. Mean ± SD, two‐tailed *t*‐test, *n* = 3. D,E) C666‐1‐A11‐LMP2A cells were cocultured with or without neutrophils in the supernatant of EBV^‐^ or EBV^+^ epithelial cancer cells for 4 h. LPM2A‐TCR‐T cells were then added, and the coculture was incubated for an additional 16 h. Surviving C666‐1‐A11‐LMP2A cells were collected and quantified by flow cytometry as PI‐ Annexin V‐ cells. Representative images (D) and analysis (E) of surviving C666‐1‐A11‐LMP2A cells are shown. Each connected pair of points represents an individual repetition of the experiment with neutrophils from different donors, and the results were compared using a ratio paired *t*‐test. *n* = 4 donors. F,G) Representative images (F) and analysis (G) of granzyme B expressed by LPM2A‐TCR‐T cells in the coculture system as depicted in (D). Each connected pair of points represents an individual repetition of the experiment with neutrophils from different donors, and the results were compared using a ratio paired *t*‐test. *n* = 6 donors. H,I) Representative images (G) and analysis (I) of IFN‐γ expressed by LPM2A‐TCR‐T cells in the coculture system as depicted in (D). Each connected pair of points represents an individual repetition of the experiment with neutrophils from different donors, and the results were compared using a ratio paired *t*‐test. *n* = 6 donors. NPC: nasopharyngeal cancer. EBVaGC: EBV‐associated gastric cancer. TCR‐T cells: LPM2A‐TCR‐T cells. PBNs: peripheral blood neutrophils. ^*^ *p* < 0.05, ^**^ *p* < 0.01, ^***^ *p* < 0.001, and ^****^ *p* < 0.0001, n.s., not significant.

To understand the role of activated TANs on CTLs‐mediated antitumor activity in EBV‐associated epithelial cancers, granzyme B expression in the infiltrating CD8^+^ T cells around the TANs was further confirmed by immunofluorescence staining. It was shown that the greater the number of MPO^+^ neutrophils that infiltrated, the fewer CD8^+^ granzyme B^+^ T cells were detected in EBV‐associated epithelial cancers (**Figure** **2**A; Figure S2A,B, Supporting Information). Nearest neighbor analysis demonstrated that in EBV‐associated epithelial cancers, in regions with high expression of MPO, CD8⁺ granzyme B⁺ cells are located farther away from tumor cells. While, in areas with low expression of MPO, CD8⁺ granzyme B⁺ cells are situated closer to tumor cells (Figure 2B,C). What's more, proximity analysis demonstrated that within 15 µm of tumor cells, there were more CD8⁺ granzyme B⁺ cells in areas with low expression of MPO than that in regions with high expression of MPO in EBV‐associated epithelial cancers. As the distance from tumor cells increased, the differences between them became nonsignificant (Figure S2C,D, Supporting Information). These results indicated that the dysfunction of CD8^+^ T cells may be caused by TANs in a contact‐dependent manner.

To verify this speculation, we carried out T lymphocyte‐mediated cytotoxicity assay in EBV‐associated epithelial cancers in vitro. We first constructed a model of C666‐1‐A11‐LPM2A cells and LMP2A‐specific T‐cell receptor engineered T cells (LPM2A‐TCR‐T cells) that express TCRs (AU011) capable of directly recognizing and targeting the C666‐1‐A11‐LPM2A cell antigen (A11‐LMP2A).^[^ ^7^ ^]^ C666‐1‐A11‐LMP2A cells were cocultured with LPM2A‐TCR‐T cells with or without neutrophils, which were treated with the supernatants derived from EBV‐positive or EBV‐negative epithelial cancer cells in advance. The results revealed that LPM2A‐TCR‐T cells were cytotoxic to tumor cells and that this effect could be inhibited by neutrophils treated with the supernatants, especially the supernatants of EBV‐positive epithelial cancer cells (Figure 2D,E), indicating that the T lymphocyte‐mediated cytotoxicity in EBV‐associated epithelial cancers was hindered by TANs. Considering that neutrophils themselves have a pro‐ or antitumor effect on cancer cells,^[^ ^33^ ^]^ C666‐1‐A11‐LMP2A cells were cocultured with neutrophils treated with the supernatants of EBV‐positive or EBV‐negative epithelial cancer cells to exclude neutrophils’ effect. The results revealed nonsignificant differences among the different groups (Figure S2E,F, Supporting Information), suggesting that there was no direct effect of neutrophils on cancer cells. T lymphocyte‐mediated cytotoxicity is largely dependent on degranulation and functional cytokine secretion, and thus we tested the expression of granzyme B and interferon γ (IFN‐γ) in LPM2A‐TCR‐T cells in the coculture system. The expressing of granzyme B or IFN‐γ in LPM2A‐TCR‐T cells decreased when these cells were cocultured with neutrophils in the supernatants of EBV‐positive epithelial cancer cells compared with the supernatants of EBV‐negative epithelial cancer cells (Figure 2F–I). Intriguingly, we found that in the supernatants of EBV‐positive epithelial cancer cells, with the addition of neutrophils, the surviving tumor cells increased while the expression of granzyme B and INF‐γ in LPM2A‐TCR‐T cells didn't show a corresponding reduction (Figure 2D–I). Therefore, we speculated that TANs in EBV‐associated epithelial cancer were induced to interfere with CTLs‐mediated cytotoxicity in a way that did not impair the function of LPM2A‐TCR‐T cells directly.

### 2.3 TANs Interfere with CTLs‐Mediated Cytotoxicity via NETs In Vitro

**Figure 3:**
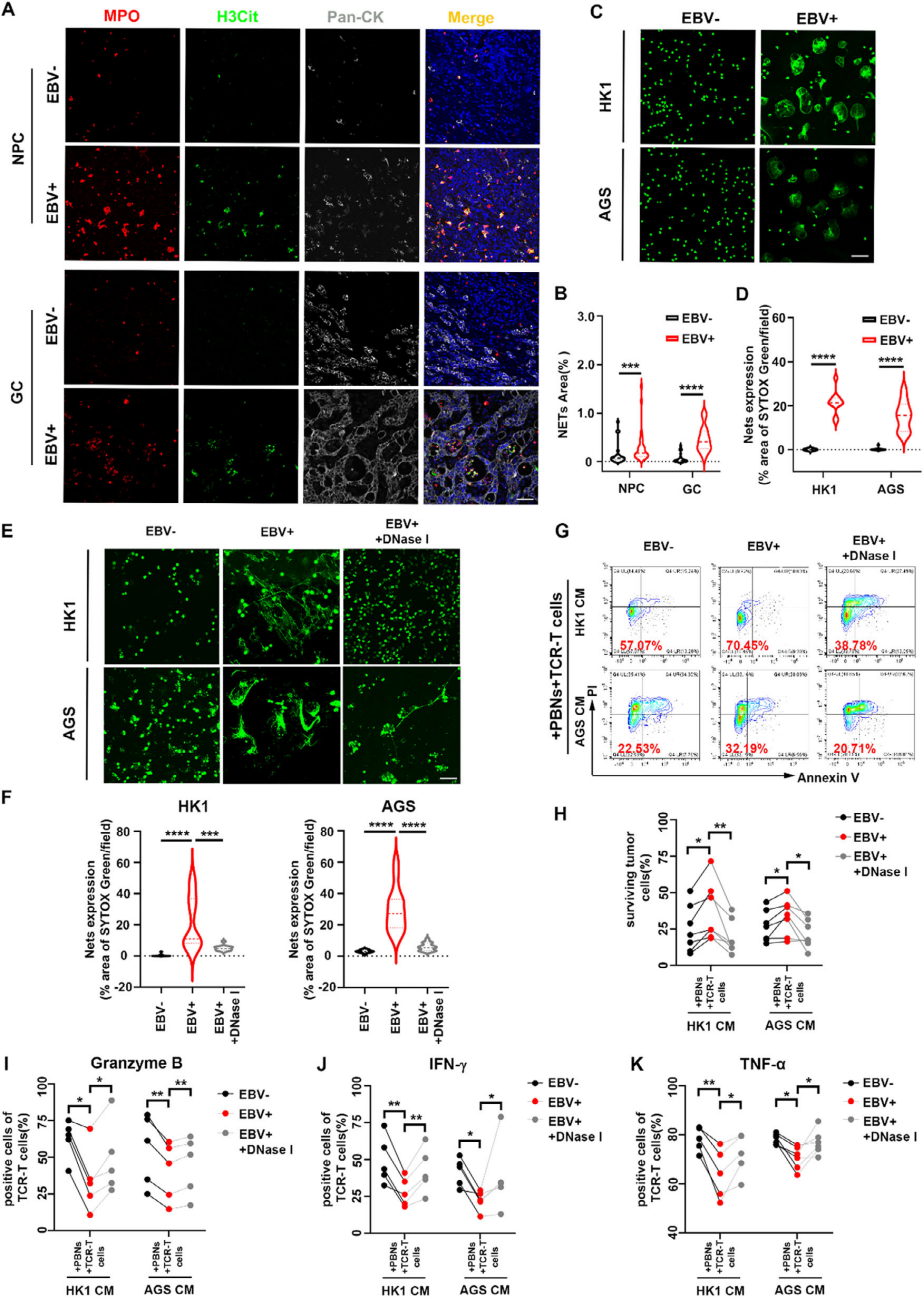
TANs interfere with CTLs‐mediated cytotoxicity via NETs in vitro. A,B) Representative single‐stained and merged images of NPC (upper panel) and GC (lower panel) sections subjected to immunofluorescence staining for MPO (red), H3Cit (green), PAN‐CK (gray), and DAPI. NETs (yellow) were identified by the co‐expression of MPO and H3Cit. Magnification: 400x. Scale bar: 50 µm. B) Statistical analysis of NETs expression in EBV^‐^ or EBV^+^ NPC and EBV^‐^ or EBV^+^ GC. Mann–Whitney *U* test. *n* = 3/4. C) Representative images of NETs formation from freshly isolated neutrophils cultured in different supernatants. Only structures positive for SYTOX Green (green) staining and depicting NETs morphology were identified as NETs. Magnification: 400x Oil. Scale bar: 50 µm. D) Statistical analysis of NETs expression in (C). Mann–Whitney *U* test. *n* = 3 donors. E,F) Representative images (E) and quantification (F) of NETs formation from neutrophils cultured in different supernatants with or without DNase I (0.5 U). Magnification: 400x Oil. Scale bar: 50 µm. Mann–Whitney *U* test. *n* = 3 donors. G,H) C666‐1‐A11‐LMP2A cells were cocultured with neutrophils in the indicated supernatant (DNase I, 0.5 U) for 4 h and then cocultured with LPM2A‐TCR‐T cells for 16 h. Surviving tumor cells were quantified by flow cytometry as PI‐ Annexin V‐ cells (G). Each connected pair of points represents an individual repetition of the experiment with neutrophils from different donors, and the results were compared using a ratio paired *t*‐test (H). *n* = 7 donors. I–K) Granzyme B (I), IFN‐γ (J) and TNF‐α (K) expressed in LPM2A‐TCR‐T cells from the T lymphocyte‐mediated cytotoxicity assay, as in (G), were tested by flow cytometry and analyzed. Each connected pair of points represents an individual repetition of the experiment with neutrophils from different donors, and the results were compared using a ratio paired *t*‐test. *n* = 5 donors. NPC: nasopharyngeal cancer. GC: gastric cancer. TCR‐T cells: LPM2A‐TCR‐T cells. PBNs: peripheral blood neutrophils. ^*^ *p* < 0.05, ^**^ *p* < 0.01, ^***^ *p* < 0.001, and ^****^ *p* < 0.0001, n.s., not significant.

It is reported that neutrophil extracellular traps (NETs) could work as a physical protection for tumor cells by interfering with immune cell‐mediated cytotoxicity.^[^ ^28^ ^]^ Thus, we wondered whether NETs formation occurs in EBV‐associated epithelial cancers and whether NETs protect tumor cells from CTLs‐mediated cytotoxicity. We first examined the formation of NETs in EBV‐associated epithelial cancers by conducting immunofluorescence staining for MPO and citrullinated histone H3 (H3Cit).^[^ ^35^ ^]^ It was shown that NETs formation was higher in EBV‐positive epithelial cancers than in EBV‐negative epithelial cancers (**Figure** **3**A,B). Next, we performed NETs formation experiment in vitro and found that neutrophils were more frequently observed to explode and extrude NETs when cultured in the supernatants of EBV‐positive epithelial cancer cells than in those of EBV‐negative epithelial cancer cells (Figure 3C,D; Figure S3A–C and Videos S1–S8, Supporting Information). DNase I is thought to digest NETs.^[^ ^36^ ^]^ When DNase I was present, NETs were diminished (Figure S3A–C and Videos S1–S8, Supporting Information; Figure 3E,F). To examine the effect of NETs on CTLs‐mediated cytotoxicity in EBV‐associated epithelial cancers, we repeated T lymphocyte‐mediated cytotoxicity assay in the presence or absence of DNase I. We found that in the presence of DNase I, the cytotoxicity of LPM2A‐TCR‐T cells was restored in the supernatants of EBV‐positive epithelial cancer cells (Figure 3G,H). We further detected the functional degranulation and cytokine production of LPM2A‐TCR‐T cells when DNase I was added. Compared with the groups of neutrophils in the supernatants of EBV‐positive epithelial cancer cells, LPM2A‐TCR‐T cells in the groups with the addition of DNase I exhibited significantly enhanced expression of granzyme B, IFN‐γ, tumor necrosis factor α (TNF‐α) and perforin (Figure S3D–G, Supporting Information; Figure 3I–K). All these results suggested that NETs formed by TANs played an important role in interfering with CTLs‐mediated cytotoxicity in EBV‐associated epithelial cancers.

### 2.4 TANs Interfere with CTLs‐Mediated Antitumor Activity via NETs In Vivo

**Figure 4:**
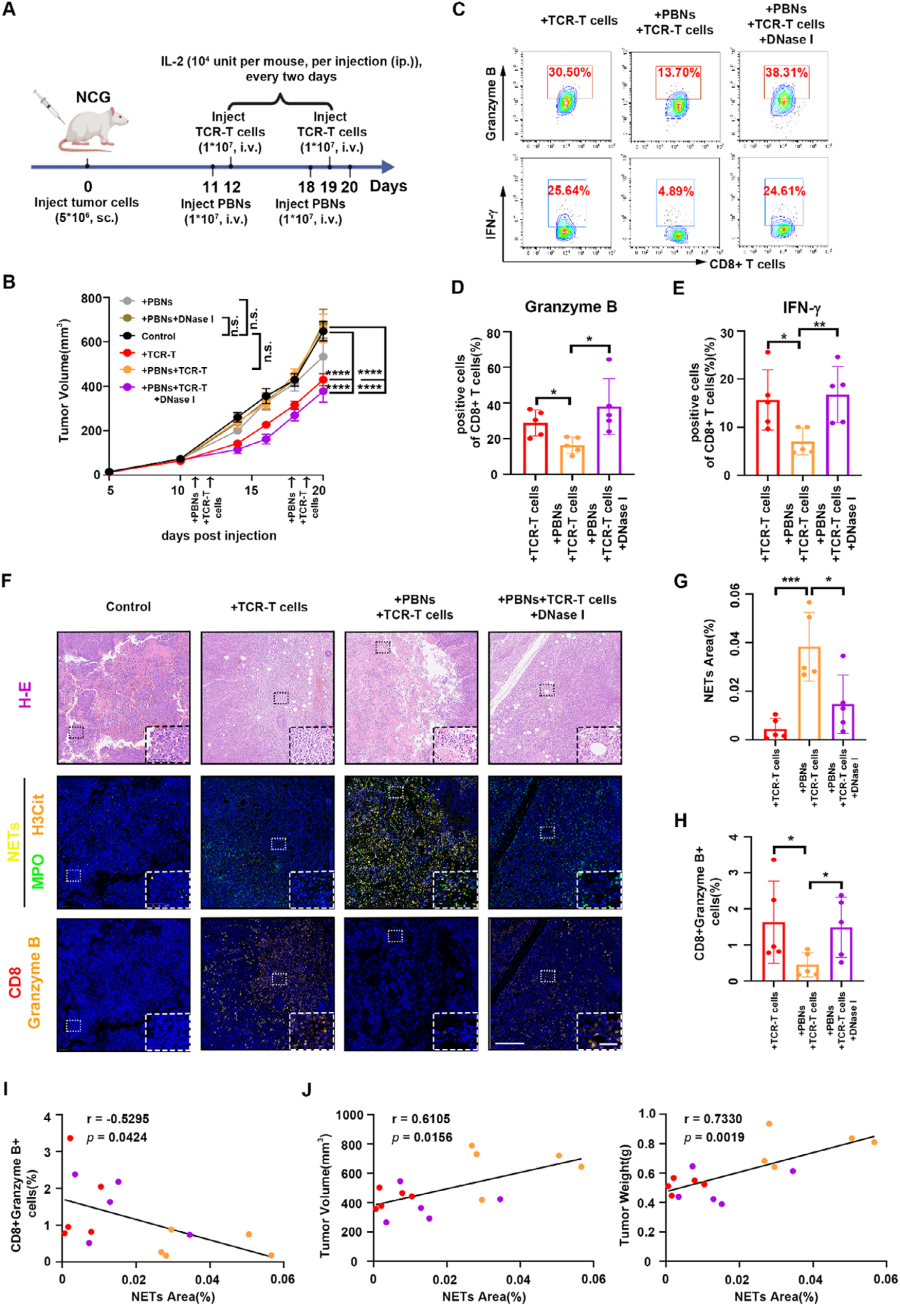
TANs interfere with CTLs‐mediated antitumor activity via NETs in vivo. A) Schematic diagram of neutrophils and/or LPM2A‐TCR‐T cells injection in the subcutaneous C666‐1‐A11‐LMP2A xenograft tumor model used for (B–E). B) Growth curves of C666‐1‐A11‐LMP2A xenograft tumors. two‐way ANOVA. *n* = 5. C–E) Tumors from C666‐1‐A11‐LMP2A xenograft‐bearing mice were collected, ground into single cells and tested for the expression of granzyme B (C, D) and IFN‐γ (C, E) in CD8^+^ T cells. *n* = 5. F) Representative merged images of serial tumor sections from different C666‐1‐A11‐LMP2A xenograft‐bearing mice. Tumor sections were subjected to immunofluorescence staining for (H–E), MPO (green) and H3Cit (orange), CD8 (red), and granzyme B (orange). NETs (yellow) were identified by the co‐expression of MPO and H3Cit. Magnifications: 40x (solid line frame), 400x (dashed line frame). Scale bars: 300 µm (40x images), 60 µm (400x images). G) NETs area in serial tumor sections from (F) was analyzed using HALO software. *n* = 5. H) The number of CD8^+^ granzyme B^+^ T cells expressed in serial tumor sections from (F) was analyzed using HALO software. *n* = 5. I) Correlation analysis between NETs and CD8^+^ granzyme B^+^ T cells detected in (F). Red, orange, and purple dots represent tumor sections from mice injected with LPM2A‐TCR‐T cells, LPM2A‐TCR‐T cells plus neutrophils, and LPM2A‐TCR‐T cells plus neutrophils plus DNase I, respectively. *n* = 15. J) Correlation analysis between NETs formation and the volume (left panel) or weight (right panel) of tumors from C666‐1‐A11‐LMP2A xenograft‐bearing mice. Red, orange, and purple dots represent tumor sections from mice injected with LPM2A‐TCR‐T cells, LPM2A‐TCR‐T cells plus neutrophils, and LPM2A‐TCR‐T cells plus neutrophils plus DNase I, respectively. *n* = 15. TCR‐T cells: LPM2A‐TCR‐T cells. PBNs: peripheral blood neutrophils. Mean ± SD are shown for all panels, including error bars. *p*‐values were calculated with Mann–Whitney *U* tests or two‐tailed *t*‐test unless indicated. ^*^ *p* < 0.05, ^**^ *p* < 0.01, ^***^ *p* < 0.001, and ^****^ *p* < 0.0001, n.s., not significant.

To further explore whether neutrophils in EBV‐associated epithelial cancer cells inhibit CTLs‐mediated cytotoxicity via NETs in vivo, we initially confirmed the capacity of C666‐1‐A11‐LMP2A cells to induce NETs formation both in vitro (Figure S4A,B, Supporting Information) and in vivo (Figure S4C,D, Supporting Information). Afterward, C666‐1‐A11‐LMP2A xenograft‐bearing mice were treated with freshly isolated neutrophils from healthy donors and LPM2A‐TCR‐T cells (**Figure** **4**A). LPM2A‐TCR‐T cells demonstrated antitumor efficacy against C666‐1‐A11‐LMP2A tumors, but this efficacy was hindered when neutrophils were present. When DNase I was injected along with the neutrophils, the immunotherapeutic efficacy of LPM2A‐TCR‐T cells was restored (Figure S4E,F, Supporting Information; Figure 4B). Neutrophils exhibited no significant efficacy against C666‐1‐A11‐LMP2A tumors, consistent with the results of experiments in vitro (Figures S2E,F and S4E,F, Supporting Information; Figure 4B). CD8^+^ T cells were extracted from tumors and analyzed for the expression of granzyme B and IFN‐γ by flow cytometry. Compared with mice treated with LPM2A‐TCR‐T cells alone, those treated with LPM2A‐TCR‐T cells and neutrophils exhibited lower expression of granzyme B and IFN‐γ in CD8^+^ T cells. However, this reduced expression was reversed in mice treated with LPM2A‐TCR‐T cells, neutrophils and DNase I (Figure 4C–E). Tumors from different mice were stained for NETs, as well as CD8 and granzyme B. The stained sections revealed a significantly higher abundance of NETs in tumors derived from mice that received co‐injections of LPM2A‐TCR‐T cells and neutrophils, compared with tumors from mice injected solely with LPM2A‐TCR‐T cells or from mice that received LPM2A‐TCR‐T cells and neutrophils along with DNase I (Figure 4F,G). Correspondingly, fewer CD8^+^ granzyme B^+^ T cells were present in tumors derived from mice that received co‐injections of LPM2A‐TCR‐T cells and neutrophils, compared with tumors from mice injected solely with LPM2A‐TCR‐T cells or from mice that received LPM2A‐TCR‐T cells and neutrophils along with DNase I (Figure 4F,H). To assess the relationship between NETs formation and the function of CD8^+^ T cells, we performed a correlation analysis and found that the NETs formation was negatively correlated with granzyme B expression in CD8^+^ T cells (Figure 4I). This negative correlation was further confirmed by flow cytometry, which demonstrated that the number of CD8^+^ granzyme B^+^ T cells and CD8^+^ IFN‐γ^+^ T cells decreased when NETs formation increased (Figure S4G, Supporting Information). Both the volume and the weight of tumors from mice in different groups were no doubt negatively related to granzyme B and IFN‐γ expression in CD8^+^ T cells (Figure S4H,I, Supporting Information). The volume and the weight of tumors were found to be positively related to NETs formation (Figure 4J). Collectively, NETs released by TANs can hamper CTLs‐mediated antitumor activity against EBV‐associated epithelial cancer cells and promote tumor growth.

### 2.5 The EBV‐Mediated CXCL10‐CXCR3 Axis Facilitates NETs Formation

To clarify the mechanism underlying NETs formation in EBV‐associated epithelial cancer cells, we employed an RNA‐sequencing (RNA‐seq) assay. A total of 116 differentially expressed genes (DEGs) were identified as consistently upregulated in the EBV‐positive epithelial cancer lines compared with the EBV‐negative epithelial cancer lines (Figure S5A, Supporting Information). Gene Ontology (GO) functional classification using the DAVID site revealed that these DEGs were involved primarily in the response to viruses, the inflammatory response, neutrophil chemotaxis and pathways related to NETs formation^[^ ^31^ ^]^ (Figure S5B, Supporting Information). Using Cytoscape software, we visualized the protein‐protein interaction (PPI) networks for these DEGs and identified core genes and biological processes. The top 10 hub genes (CXCL10, CCL5, IAGAM, ITGAX, CSF1, OSA2, BST2, IFIT3, RSAD2, and NFKFBIA) were identified, and only CXCL10 and CCL5 were implicated in both the pathways of viral response and granulocyte chemotaxis (Figure S5C, Supporting Information). CXCL10 and CCL5 were then confirmed to be expressed at significantly higher levels in EBV‐positive epithelial cancer cells than in EBV‐negative epithelial cancer cells (Figure S5D,E, Supporting Information). Considering that CXCL10 and CCL5 have been reported to regulate immune cell migration,^[^ ^37^, ^38^ ^]^ we first applied a chemotaxis assay to assess the ability of CXCL10 and CCL5 to regulate neutrophil migration. It was shown that CXCL10 or CCL5 exhibited chemotactic effect on neutrophils when its concentration reached 100 ng mL^−1^. However, this effect was relatively minor when compared with that elicited by CXCL8 (Figure S5F, Supporting Information). What's more, the supernatants of EBV‐positive epithelial cancer cells exhibited a significantly greater capacity to attract neutrophils compared to those of EBV‐negative epithelial cancer cells. In the supernatants of HK1 cells, the addition of CXCL10 or CCL5 did not elicit any significant effect on neutrophil recruitment. However, in the supernatant of AGS cells, the addition of CXCL10 and CCL5 demonstrated a notable chemotactic effect on neutrophils. CXCR3 and CCR5 are receptors for CXCL10 and CCL5, respectively.^[^ ^37^, ^38^ ^]^ Therefore, AMG487 (an antagonist of CXCR3) and maraviroc (an antagonist of CCR5) were added to the supernatants of EBV‐positive epithelial cancer cells. When compared with the supernatants of AGS‐EBV cells, a significant reduction in the number of neutrophils attracted was observed upon the addition of AMG487 alone or in combination with maraviroc. However, this reduction was not statistically significant in the supernatants of HK1‐EBV cells (Figure S5G,H, Supporting Information). These results indicated that EBV‐associated epithelial cancer cells exerted a strong chemotactic effect on neutrophils, with CXCL10 and CCL5 likely playing a role in this process.

**Figure 5:**
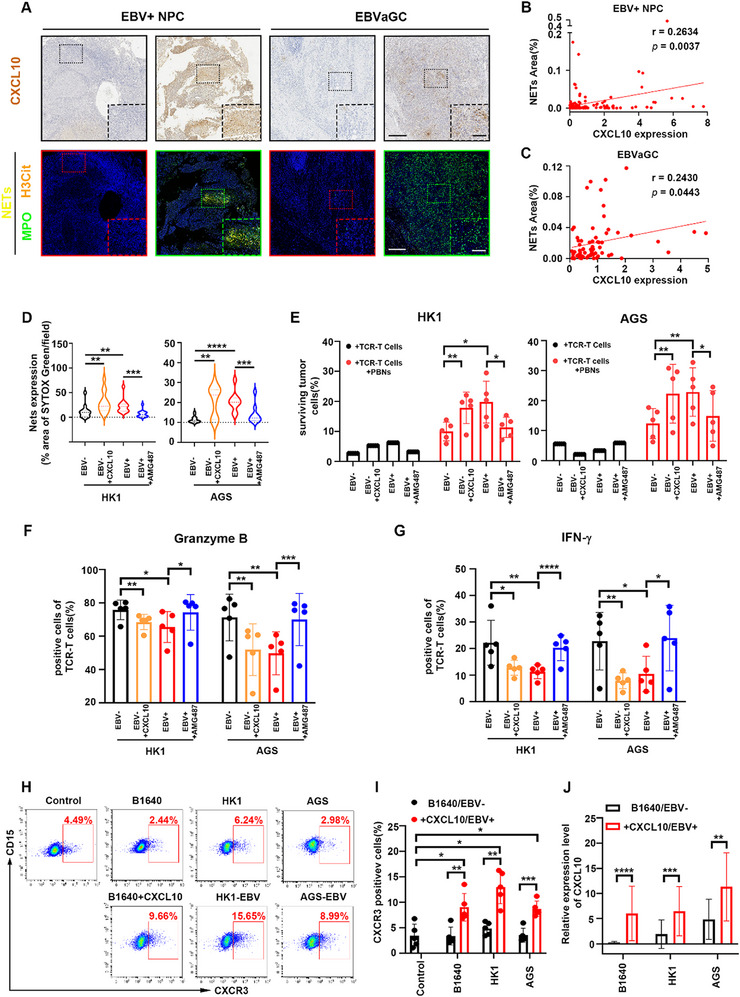
The EBV‐mediated CXCL10‐CXCR3 axis facilitates NETs formation. A) Representative images of serial tumor sections from EBV^+^ NPC (left panel) and EBVaGC (right panel). Tumor sections were subjected to immunohistochemistry staining for CXCL10 and immunofluorescence staining for MPO (green) and H3Cit (orange). NETs (yellow) were identified by the co‐expression of MPO and H3Cit. The red line frames represent areas with low CXCL10 expression and low NETs levels; the green line frames represent areas with high CXCL10 expression and high NETs levels. Magnifications: 40x (solid line frames), 200x (dashed line frames). Scale bars: 250 µm (40x images), 100 µm (200x images). B,C) Correlation analysis between CXCL10 expression and NETs expression in EBV^+^ NPC (B) and in EBVaGC (C). The analysis was conducted on the basis of 120 EBV^+^ NPC samples and 69 EBV^+^ GC samples. D) Quantification of NETs formed by freshly isolated neutrophils cultured in the supernatants of EBV^−^ and EBV^+^ epithelial cancer cells with or without CXCL10 (200 ng mL^−1^) or AMG487 (2 µm) for 4 h. *n* = 3. E) C666‐1‐A11‐LMP2A cells were cocultured with neutrophils for 4 h and LPM2A‐TCR‐T cells for additional 16 h in the indicated supernatants. Analysis of surviving C666‐1‐A11‐LMP2A cells tested by flow cytometry. Each dot represents an individual repetition of the experiment with neutrophils from different donors, and the results were compared using a ratio paired *t*‐test. *n* = 5 donors. F,G) Analysis of granzyme B (F) and IFN‐γ (G) expressed by LPM2A‐TCR‐T cells from the coculture system in (E). Each dot represents an individual repetition of the experiment with neutrophils from different donors, and the results were compared using a ratio paired *t*‐test. *n* = 5 donors. H,I) Representative images (H) and analysis (I) of CXCR3 expression on neutrophils freshly isolated (control), or after cultured in RPMI 1640 (B1640) supplemented with or without CXCL10 (200 ng mL^−1^), and the supernatants of EBV^‐^ and EBV^+^ epithelial cancer cells for 4 h. Each dot represents an individual repetition of the experiment with neutrophils from different donors, and the results were compared using a ratio paired *t*‐test. *n* = 5 donors. J) Neutrophils cultured as in (H) were collected and tested for CXCL10 expression. *n* = 3 donors. NPC: nasopharyngeal cancer. EBVaGC: EBV‐associated gastric cancer. TCR‐T cells: LPM2A‐TCR‐T cells. PBNs: peripheral blood neutrophils. Mean ± SD are shown for all panels, including error bars. *p*‐values were calculated with Mann–Whitney *U* test or two‐tailed *t*‐test unless indicated. ^*^ *p* < 0.05, ^**^ *p* < 0.01, ^***^ *p* < 0.001, and ^****^ *p* < 0.0001, n.s., not significant.

Next, we aimed to test how CXCL10 and/or CCL5 influence the extrusion of NETs. We added CXCL10 or CCL5 to RPMI 1640 medium and performed a NETs formation assay, which revealed that the addition of CXCL10 induced NETs formation (Figure S6A,B, Supporting Information). The introduction of GSK484, a PAD4 inhibitor, was confirmed to effectively suppress the formation of NETs triggered by CXCL10 (Figure S6C–F, Supporting Information). These results indicated that CXCL10 induced the process of NETosis. What's more, we analyzed the correlation between CXCL10 expression and NETs formation in 120 EBV‐associated NPC samples and 69 EBVaGC samples. It was found that NETs formed in EBV‐positive NPC (**Figure** **5**A,B) and in EBVaGC (Figure 5A,C) were positively correlated with CXCL10 expression. Further studies demonstrated that CXCL10 can induce NETs formation, which subsequently disrupt the contact between LPM2A‐TCR‐T cells and tumor cells (Figure S7A, Supporting Information). The cytotoxicity assay showed that the introduction of CXCL10 had no detrimental effect on either tumor cells or LPM2A‐TCR‐T cells, but its presence significantly influenced the cytotoxicity of LPM2A‐TCR‐T cells when neutrophils were in the coculture system (Figure S7B,C, Supporting Information). To elucidate the role of CXCL10 in NETs formation induced by EBV‐positive epithelial cancer cells, we constructed CXCL10‐knockdown cell lines and confirmed the decreased expression of CXCL10 (Figure S8A,B, Supporting Information). We applied the supernatants of CXCL10‐knockdown cell lines, the anti‐CXCL10 antibody, GSK484 (PAD4 inhibitor), N‐Acetyl‐L‐cysteine (ROS inhibitor), and apocynin (NADPH oxidase complex inhibitor) in the NETs formation assay. Compared with the supernatants of EBV‐positive epithelial cancer cells, both CXCL10 knockdown and CXCL10 blockade significantly inhibited the formation of NETs. The same change occurred following the addition of GSK484, N‐Acetyl‐L‐cysteine or apocynin (Figure S8C–E, Supporting Information), suggesting that CXCL10, PAD4, ROS and the NADPH oxidase complex were involved in NETs formation induced by EBV‐positive epithelial cancer cells.

We then sought to determine whether CXCR3 was involved in this process. The SYTOX assay was performed by adding CXCL10 to the supernatants of EBV‐negative epithelial cancer cells and adding AMG487 to the supernatants of EBV‐positive epithelial cancer cells. CXCL10 and AMG487 were found to function in a dose‐dependent manner (Figure S9A,B, Supporting Information). Furthermore, it was confirmed that the addition of CXCL10 in the supernatants of EBV‐negative epithelial cancer cells induced NETs formation, and CXCR3 blockade hampered the ability of the supernatants of EBV‐positive epithelial cancer cells to induce NETs formation (Figure S9C, Supporting Information; Figure 5D). We then repeated the T lymphocyte‐mediated cytotoxicity assay with the addition of CXCL10 and AMG487. The results revealed that when CXCL10 was added to the coculture system of C666‐1‐A11‐LMP2A cells, LPM2A‐TCR‐T cells and neutrophils in the supernatants of EBV‐negative epithelial cancer cells, the cytotoxicity of LPM2A‐TCR‐T cells was inhibited, and when AMG487 was added to the coculture system in the supernatants of EBV‐positive epithelial cancer cells, the cytotoxicity of LPM2A‐TCR‐T cells was significantly enhanced (Figure S9D,E, Supporting Information; Figure 5E). The functional degranulation and cytokine release in LPM2A‐TCR‐T cells were assessed. Compared with the supernatants of EBV‐negative epithelial cancer cells, the expression of granzyme B, IFN‐γ, TNF‐α and perforin in LPM2A‐TCR‐T cells decreased when CXCL10 was added to the coculture system. Compared with the supernatants of EBV‐positive epithelial cancer cells, the expression of granzyme B, IFN‐γ, TNF‐α and perforin in LPM2A‐TCR‐T cells increased when AMG487 was added to the coculture system (Figure S9F–I, Supporting Information; Figure 5F,G). These results led to the conclusion that the elevated CXCL10 levels in the supernatants of EBV‐negative epithelial cancer cells could induce NETs formation, and the blockade of CXCR3 in the supernatants of EBV‐positive epithelial cancer cells could suppress NETs formation, thus influencing LPM2A‐TCR‐T‐cell cytotoxicity. Next, we assessed the expression of CXCR3 on neutrophils. The freshly purified human neutrophils were observed to exhibit low‐level expression of CXCR3. After cultured in different supernatants, the expression of CXCR3 on neutrophils was found to be modulated. Specifically, compared with freshly purified human neutrophils, the expression of CXCR3 was significantly upregulated in neutrophils cultured in the supernatants of EBV‐positive epithelial cancer cells or in supernatants supplemented with CXCL10. However, neutrophils cultured in the supernatants of EBV‐negative epithelial cancer cells or in supernatants devoid of CXCL10 did not exhibit a similar increase in CXCR3 expression (Figure 5H,I). Moreover, neutrophils cultured in the supernatants of EBV‐positive epithelial cancer cells or in supernatants supplemented with CXCL10 exhibited a significant upregulation of CXCL10 compared with those cultured in the supernatants of EBV‐negative epithelial cancer cells or in supernatants devoid of CXCL10 (Figure 5J). These findings suggested that CXCL10‐CXCR3 axis in EBV‐associated epithelial cancers worked in a positive feedback loop to induce NETs extrusion.

### 2.6 The Clinical Significance of NETs in Patients with EBV‐Associated Epithelial Cancers

**Figure 6:**
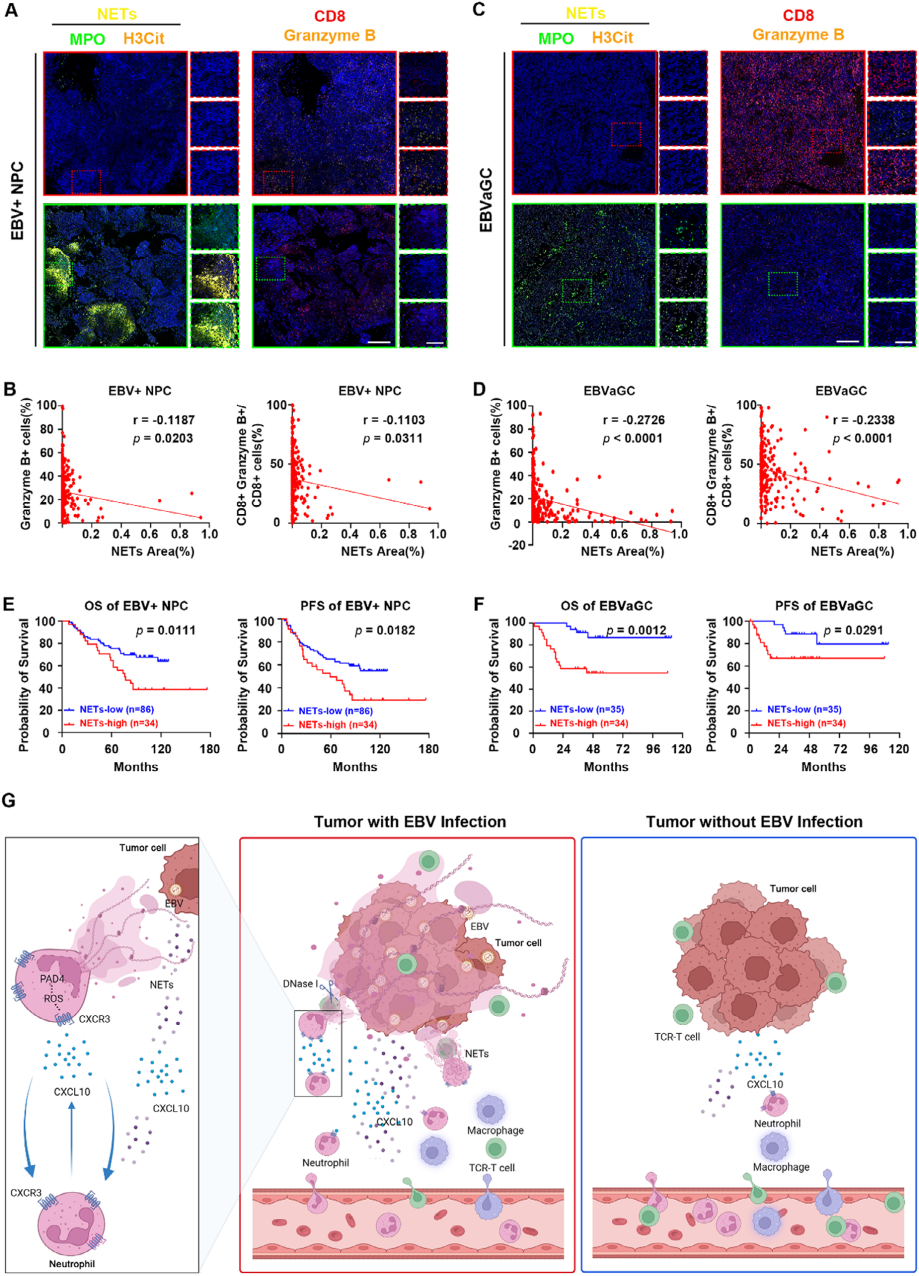
The clinical significance of NETs in patients with EBV‐associated epithelial cancers. A,C) Representative images of serial tumor sections from EBV^+^ NPC (A) and EBVaGC (C). Tumor sections were subjected to immunofluorescence staining for MPO (green) and H3Cit (orange), CD8 (red), and granzyme B (orange). NETs (yellow) were identified by the co‐expression of MPO and H3Cit. The red line frames represent areas with low NETs levels but high CD8 and granzyme B expression; the green line frames represent areas with high NETs levels but low CD8 and granzyme B expression. The solid line frames represent the merged images with a magnification of 40x; the dashed line frames represent the single‐stained and merged images with a magnification of 200x. Scale bars: 250 µm (40x images), 100 µm (200x images). B,D) Correlation analysis between NETs levels and granzyme B^+^ cells (left panel) or the ratio of CD8^+^ granzyme B^+^ cells/ CD8^+^ cells (right panel) in EBV^+^ NPC (B) and in EBVaGC (D). *p*‐values were calculated with a two‐tailed *t*‐test. E,F) All Kaplan–Meier plots show patients split into groups with high or low NETs levels. The OS and PFS of EBV^+^ NPC patients (E) and EBVaGC patients (F) were analyzed. *p*‐values were calculated with the log‐rank test. G) Schematic illustrating the major findings of this study: EBV‐associated epithelial cancer cells secrete chemokines, including CXCL10 and CCL5, to attract neutrophils. These neutrophils are induced to form abundant NETs via the CXCL10‐CXCR3 axis through a positive feedback loop. NETs formed in EBV‐associated epithelial cancers facilitate the growth of tumor cells by interfering with CTLs‐mediated cytotoxicity. NPC: nasopharyngeal cancer. EBVaGC: EBV‐associated gastric cancer. OS: overall survival. PFS: disease‐free survival.

Given that CTLs‐mediated cytotoxicity was negatively related to NETs in the abovementioned experiments, we analyzed the distribution of NETs in EBV‐associated epithelial cancers and their relationships with CTLs. The results revealed that in regions where NETs levels were elevated, the expression of granzyme B was reduced, both in EBV‐associated NPC (**Figure** **6**A,B) and in EBVaGC (Figure 6C,D). Additionally, in regions with increased NETs levels, there was a reduction in the proportion of CD8⁺ granzyme B⁺ cells among all CD8⁺ cells, indicating a negative correlation between NETs formation and CTLs‐mediated cytotoxicity. We then performed a survival analysis to explore the relationship between NETs and patient survival. The results showed that patients with EBV‐associated epithelial cancers with higher levels of NETs experienced shorter overall survival (OS) and shorter progression‐free survival (PFS) (Figure 6E,F). The multivariate Cox regression analysis revealed that NETs may be an independent prognostic indicator for patients with EBV‐associated NPC (**Table** **1**) and patients with EBVaGC (**Table** **2**). Besides, circulatory EBV‐DNA was shown to be an independent prognostic indicator for patients with EBV‐associated NPC (Table 1). Specifically, a higher load of circulatory EBV‐DNA indicated a shorter OS and a shorter PFS for patients with EBV‐associated NPC. In patients with EBVaGC, in addition to NETs, an advanced N stage was indicative for shorter OS and shorter PFS, so was metastasis for OS (Table 2). We also conducted a correlation analysis to determine whether NETs levels and various clinical‐pathological parameters were correlated. No obvious associations were found between NETs and any of these various parameters, except that elderly patients (≥60 years old) with EBVaGC held higher NETs level than younger patients did (Tables S1 and S2, Supporting Information). Altogether, high levels of NETs were found to be associated with poor T lymphocyte‐mediated immunity and a poor prognosis for patients with EBV‐associated epithelial cancers.

**Table 1:** Univariate and multivariate analysis of OS and PFS in patients with EBV+ NPC.

	EBV^+^ NPC (*n* = 120)							
Variables	Overall survival				Progression‐free survival			
	Univariate cox		Multivariate cox		Univariate cox		Multivariate cox	
	*p*‐value	HR [95%CI]	*p*‐value	HR [95%CI]	*p*‐value	HR [95%CI]	*p*‐value	HR [95%CI]
**Age(y)**								
≥50 vs <50	0.621	1.157 (0.649–2.064)			0.722	0.909 (0.538–1.537)		
**Gender**								
male vs female	0.796	1.096 (0.546–2.200)			0.212	0.699 (0.399–1.226)		
**Clinical Stage**								
IV vs II‐III	0. 158	1.512 (0.851–2.686)			0.236	1.363 (0.817–2.273)		
**T Stage**								
T3‐T4 vs T1‐T2	0.390	1.569 (0.562–4.381)			0.227	1.872 (0.678–5.170)		
**N Stage**								
N2‐N3 vs N0‐N1	0.177	1.485 (0.836–2.638)			0.056	1.657 (0.988–2.781)		
**M Stage**								
M1 vs M0	0.333	2.015 (0.488–8.317)			0.573	1.500 (0.366–6.151)		
**EBV DNA (copies/ml)**								
>8471 vs ≤8471	**0.061**	1.717 (0.974–3.026)	**0.042**	1.804 (1.022–3.185)	**0.019**	1.833 (1.104–3.046)	**0.008**	2.006 (1.200–3.356)
**NETs**								
high vs low	**0.013**	2.072 (1.165–3.685)	**0.009**	2.162 (1.213–3.853)	**0.021**	1.853 (1.099–3.124)	**0.008**	2.047 (1.205–3.477)

**Abbreviations**: CI: confident interval; NPC: Nasopharyngeal carcinoma; HR: hazard ratio; NETs: neutrophil extracellular traps; OS: overall survival; PFS: progression‐free survival.

**Table 2:** Univariate and multivariate analysis of OS and PFS in patients with EBVaGC.

	EBVaGC [*n* = 69]							
Variables	Overall survival				Progression‐free survival			
	Univariate cox		Multivariate cox		Univariate cox		Multivariate cox	
	*p*‐value	HR [95%CI]	*p*‐value	HR [95%CI]	*p*‐value	HR [95%CI]	*p*‐value	HR [95%CI]
**Age(y)**								
≥60 vs <60	0.437	1.430 (0.581–3.521)			0.712	0.817 (0.279–2.394)		
**Gender**								
male vs female	0.690	1.285 (0.374–4.414)			0.660	1.396 (0.315–6.190)		
**Clinical Stage**								
III‐IV vs I‐II	0. 079	2.686 (0.891–8.102)			0.112	2.811 (0.784–10.072)		
**T Stage**								
T3‐T4 vs T0‐T2	0.119	3.211 (0.741–13.915)			0.261	2.355 (0.528–10.499)		
**N Stage**								
N2‐N3 vs N0‐N1	**0.032**	3.354 (1.111–10.126)	**0.013**	4.211 (1.356–13.079)	**0.048**	3.674 (1.009–13.377)	**0.038**	4.071 (1.080–15.344)
**M Stage**								
M1 vs M0	**0.026**	3.500 (1.158–10.582)	**0.012**	4.451 (1.392–14.236)	0.091	3.004 (0.838–10.765)		
**HER‐2**								
high vs low	0.344	0.627 (0.238–1.650)			0.386	0.622 (0.212–1.820)		
**NETs**								
high vs low	**0.004**	5.168 (1.711–15.613)	**0.001**	7.124 (2.245–22.607)	**0.038**	3.133 (1.063–9.232)	**0.028**	3.411 (1.140–10.206)

**Abbreviations**: CI: confident interval; EBVaGC: EBV‐associated gastric carcinoma; HR: hazard ratio; NETs: neutrophil extracellular traps; OS: overall survival; PFS: progression‐free survival.

## 3 Discussion

There exists increasing evidence that EBV‐associated cancer cells can influence the TME to their own benefit by establishing an immunosuppressive TME, including Tregs, M2 macrophages and cancer‐associated fibroblasts (CAFs).^[^ ^39^ ^]^ TANs, which account for a large percentage of immune cells in the TME, have attracted little interest in EBV‐associated cancers. Here, we propose that in EBV‐associated cancers, TANs play an important part in forming an immunosuppressive TME by extruding NETs.

A recent report has found that EBV‐associated T/NK cell diseases exhibited large expansions of myeloid‐derived suppressor cells that may suppress the antiviral T‐cell response.^[^ ^40^ ^]^ Similarly, our study has found that a large abundance of neutrophils, which are derived from myeloid‐derived suppressor cells, were present in EBV‐associated cancers and were negatively correlated with the cytotoxic T‐cell response. Besides, we found that more CD8^+^ T cells were attracted to EBV‐associated tumors than to EBV‐negative tumors, in agreement with previous studies.^[^ ^5^, ^6^ ^]^ TANs possess a wide array of functions, among which NETs have the potential to promote tumor metastasis and progression.^[^ ^41^ ^]^ Our study has elucidated that NETs facilitated tumor progression by hindering the killing capacity of LPM2A‐TCR‐T cells in EBV‐associated cancers both in vitro and in vivo. NETs are a network of DNA, histones, proteases, and other cytotoxic and highly inflammatory compounds, including myeloperoxidase (MPO) and neutrophil elastase. NET‐DNA reportedly impairs CD8^+^ T‐cell immunity by binding to TMCO6 on T cells^[^ ^29^ ^]^ and promotes cancer metastasis by binding to CCDC25 on tumor cells.^[^ ^26^ ^]^ During NETosis, ROS is released and is thought to suppress CD8^+^ T cells.^[^ ^42^ ^]^ Besides, NETs are reported to impair the contact of immune cytotoxic cells with tumor cells, therefore promoting tumor progression. Hence, we wondered whether NETs formed in EBV‐positive cancers exert their effect via the physical protection of tumor cells from CTLs or through the direct suppressive effects of NET‐bound materials on CTLs. We implemented DNase I, which is believed to digest the DNA structure of NETs, both in vitro and in vivo. It turned out that once the structure of NETs was destroyed, the number of surviving tumor cells decreased, implicating the restored cytotoxicity of LPM2A‐TCR‐T cells. These findings indicate that the immunosuppressive effect of NETs in EBV‐associated cancers primarily depends on their physical interference with CTLs.

In this study, NETs were abundant in EBV‐associated cancers, as determined by an analysis of clinical specimens and cytological analysis, suggesting that NETs elicitor(s) may exist in the TME of EBV‐associated cancers. Virus infection has been found to be closely related to NETs formation in previous studies.^[^ ^36^ ^]^ Viruses were reported to trigger NETs formation by directly binding to receptors on neutrophils, such as Toll‐like receptors (TLRs) TLR7 and TLR8.^[^ ^43^, ^44^ ^]^ Respiratory syncytial virus was thought to induce NETs formation by secreting viral proteins that bind to TLR4 on neutrophils.^[^ ^45^ ^]^ Hepatocellular carcinoma with HBV infection was reported to secret S100A9 that bond to TLR4 on neutrophils and induced NETs formation.^[^ ^46^ ^]^ However, the relationship between EBV infection and NETs formation has not been investigated. In this study, we observed an increase in S100A9 expression in EBV‐positive cancer cells and explored its effect on NETs formation, but the effect was not obvious (data not shown). Through RNA sequencing, we identified two neutrophil chemoattractants, CXCL10 and CCL5, that were upregulated in EBV‐positive cancer cells. This result is in accord with the finding in a single‐cell transcriptomic analysis.^[^ ^47^, ^48^ ^]^ A recent report showed that NETs could be induced by neutrophil chemoattractant, CXCL1‐CXCL8, which interact with CXCR1 and CXCR2 with high affinity.^[^ ^28^ ^]^ CXCL10, a member of the CXC chemokine family, was found to induce NETs formation in this study. We also found that when CXCR3 was blocked, the effect of CXCL10 on NETs formation was hampered. These phenomena may suggest that the CXCR3 is involved in NETs formation. In addition to CXCL10, CXCL9 and CXCL11 are also chemokines that bind to the CXCR3 receptor.^[^ ^38^ ^]^ Whether these two chemokines have an effect on NETs extrusion needs further investigation. CXCR3 was believed to be preferentially expressed on T lymphocytes, natural killer (NK) cells, monocytes and dendritic cells, but not on naïve neutrophils.^[^ ^38^ ^]^ In our study, we found a low‐level expression of CXCR3 on freshly isolated human neutrophils. Several studies have reported CXCR3 expression on neutrophils from patients with chronic inflammatory lung diseases and rheumatoid arthritis,^[^ ^49^ ^]^ and from mice treated with acid or influenza virus.^[^ ^50^ ^]^ In agreement with these findings, our study revealed increased expression of CXCR3 on neutrophils cultured in supernatants of EBV‐associated epithelial cancer cells or in CXCL10‐supplemented supernatant. What's more, neutrophils cultured in these supernatants exhibited upregulated expression of CXCL10. Previous research findings have suggested the existence of an autocrine regulatory effect between CXCL10 and CXCR3.^[^ ^51^ ^]^ The CXCL10‐CXCR3 signaling axis was believed to promote the expression of CXCL10 and CXCR3 by modulating the NF‐κB signaling pathway.^[^ ^52^, ^53^, ^54^ ^]^ Moreover, researchers have identified a positive feedback loop involving CXCL10‐CXCR3‐IFN‐γ in cytotoxic T cells. The binding of CXCL10 to CXCR3 on T cells enhanced the production of IFN‐γ, which in turn stimulated the expression of CXCL10 and CXCR3.^[^ ^55^ ^]^ Therefore, based on the experimental results of this study, we have proposed that the upregulated CXCL10 in EBV‐positive tumor cells, upon binding to CXCR3 on neutrophils, not only stimulated the formation of NETs but also exerted a positive feedback effect, promoting higher expression of CXCL10 and CXCR3 in neutrophils. In addition, we have found that PAD4, ROS and the NADPH oxidase complex were involved in the EBV‐associated epithelial cancer cell‐mediated NETs formation. However, the mechanisms by which EBV upregulates CXCL10 expression remain to be elucidated and represent crucial areas for future research endeavors.

Emerging evidence has demonstrated a prognostic role for NETs in various types of tumors,^[^ ^56^ ^]^ such as hepatocellular carcinoma^[^ ^29^ ^]^ and colon cancer,^[^ ^57^ ^]^ predicting a poor prognosis. While data regarding the correlation between NETs and EBV‐associated epithelial cancers is scarce, our study has illustrated that NETs in tumor sections were positively correlated with shorter OS and shorter PFS of patients with EBV^+^ NPC or EBVaGC, which is consistent with results obtained from other cancers. NETs have been reported to be closely related to metastasis,^[^ ^31^ ^]^ especially in patients with lung cancer, breast cancer or hepatocellular carcinoma. In our study, no significant relationship was observed between NETs and metastasis of either EBV^+^ NPC or EBVaGC. This discrepancy might be due to the intrinsic differences among different tumors. Additionally, an interesting phenomenon was observed in this study: a positive relationship was identified between NETs and aging of patients with EBVaGC. Aging is believed to be associated with high levels of activated immune cells and inflammatory markers in the circulation and in solid tissues.^[^ ^58^ ^]^ This inflammatory state in blood and solid tissues might explain the elevated level of NETs in aged patients with EBVaGC. However, whether this positive relationship is universal in other tumors requires further investigation.

All in all, our study revealed that in EBV‐associated epithelial cancers, neutrophils were attracted and induced to extrude NETs, thus interfering with LPM2A‐TCR‐T‐cell cytotoxicity and promoting tumor progression. This process was verified to be stimulated by the CXCL10‐CXCR3 axis through a positive feedback loop. In addition, NETs were found to be negatively associated with CTLs‐mediated cytotoxicity and were identified to be an independent biomarker for predicting the prognosis of patients with EBV‐associated epithelial cancers.

## 4 Experimental Section

### Sample Collection

All patient samples were obtained from Sun Yat‐Sen University Cancer Center. For immunohistochemical and immunofluorescence staining, 120 paraffin‐embedded tissues were collected from patients with primary nasopharyngeal carcinoma (NPC), and 69 paraffin‐embedded tissues were collected from patients with primary gastric cancer (GC). These tissues were sliced and confirmed to be EBV‐infected by pathologists. Fresh peripheral blood was obtained from healthy humans in a blood donor database provided by the Knowcell Company to isolate neutrophils. All samples in the study were confirmed by a pathologic analysis.

### Cell Culture

EBV^−^ and EBV^+^ epithelial cancer cells, namely, HK1, HK1‐EBV, AGS, and AGS‐EBV cells, were obtained from Tong Xiang, Sun Yat‐sen University Cancer Center, and cultured in complete RPMI 1640 medium (GIBCO, 10% FBS, NEZERUM). All the cell lines were cultured in a humidified incubator with 5% CO2 at 37 °C. For the collection of tumor cell supernatants, the same number of paired cells was resuspended in the same volume of complete culture medium. After 3 days of incubation, the supernatants were collected.

### Generation of LPM2A‐TCR‐T Cells

Briefly, the DNA sequence of LPM2A TCR Vα with the murine TCR Cα and LPM2A TCR Vβ with the murine TCR Cβ separating by the P2A peptide (ATNFSLLKQAGDVEENPGP) were synthesized and subcloned into the MP71 retroviral vector (TCRCure Biological Technology Company). Fresh PBMCs were isolated from peripheral blood from healthy volunteers by density gradient separation and seeded in X‐vivo medium (LONZA) supplemented with human IL‐2 (1000 IU mL^−^, Beijing Four Rings Bio‐Pharmaceutical Co. Ltd) in a 24‐well plate (1  ×  10^6 cells per well). ImmunoCult Hu CD3/CD28 TCell Act (25 µL mL^−1^, Stemcell, 10 971) were added to each well to activate T cells. After 48 h, TCR virus (supplied by TCRCure Biological Technology Company) was added to Retronectin (15 µg mL^−1^, Takara, T100A)‐coated 24‐well plates and centrifuged at 2000 rpm for 120 min at 32 °C. After centrifugation, supernatants were removed, and 5  ×  10^5 activated T cells were transferred to the plates and centrifuged at 1000 rpm for 30 min at 30 °C. After centrifugation, supernatants were removed and replaced with fresh medium containing 1000 IU mL^−1^ IL‐2. Transduction efficiency was measured in 72 h by using a specific tetramer (HLA‐A*11:01 EBV LMP2 Tetramer‐SSCSSCPLSK‐PE, TS‐M111‐1). Transduced T cells were cultured with replaced fresh medium containing 1000 IU/mL IL‐2 every 3 days, and were cryopreserved in 10 days post‐transduction.

### Generation of C666‐1‐A11‐LPM2A Cells

The DNA sequences (ATGGCCGTGATGGCTCCTAGGACACTGCTGCTGCTGCTGTCAGGAGCTCTGGCACTGACACAGACTTGGGCCGGAAGCCACAGCATGAGATACTTCTACACCAGCGTGTCCAGGCCCGGAAGAGGAGAGCCCAGATTCATCGCCGTGGGATACGTGGACGACACCCAGTTCGTGCGCTTCGATAGCGACGCCGCTAGCCAGAGAATGGAGCCTAGAGCCCCTTGGATCGAGCAGGAAGGACCCGAGTATTGGGACCAGGAGACCAGGAACGTGAAGGCTCAGAGCCAGACCGACAGAGTGGATCTGGGAACCCTGCGGGGCTACTACAACCAGAGCGAAGACGGAAGCCACACCATCCAGATCATGTACGGTTGCGACGTGGGACCAGACGGCAGATTCCTGAGAGGCTACAGGCAGGACGCTTACGACGGCAAGGACTACATCGCCCTGAACGAGGACCTGAGAAGCTGGACCGCCGCAGACATGGCAGCCCAGATCACCAAGCGCAAGTGGGAAGCAGCTCACGCAGCCGAACAGCAGAGAGCCTATCTGGAAGGCCGCTGCGTGGAATGGCTGAGACGCTACCTGGAGAACGGCAAGGAGACCCTGCAGAGAACCGACCCTCCTAAGACCCACATGACCCACCACCCCATCAGCGACCACGAAGCTACACTCCGCTGTTGGGCTCTGGGATTCTACCCAGCCGAGATCACCCTGACCTGGCAGAGAGACGGAGAGGATCAGACCCAGGACACCGAACTGGTGGAGACAAGACCAGCCGGAGACGGAACATTTCAGAAGTGGGCCGCCGTGGTGGTGCCATCAGGAGAGGAGCAGAGATACACTTGCCACGTGCAGCACGAGGGACTGCCTAAGCCTCTGACACTCCGCTGGGAACTGTCTAGCCAGCCTACCATCCCTATCGTGGGCATCATTGCCGGACTGGTGCTGCTGGGAGCAGTGATCACAGGAGCAGTGGTGGCAGCAGTCATGTGGAGGAGGAAGAGCAGCGACCGGAAGGGAGGAAGCTATACACAGGCCGCCTCTAGCGATAGCGCTCAGGGAAGCGACGTGTCTCTGACCGCTTGCAAGGTGTAATGA) encoding HLA‐A11 were synthesized and subcloned into the MSCV retroviral vector (TCRCure Biological Technology Company). The LMP2A‐expressing cell line, C666‐1, was transduced with the HLA‐A11 virus. C666‐1‐A11‐LMP2A cells were cultured in complete DMEM medium (GIBCO), 10% FBS, NEZERUM).

### Cell Transfections

Short interfering RNAs (shRNAs) specifically targeting CXCL10 were synthesized and then used to generate lentiviruses by Obio Technology (Shanghai, China). HK1‐EBV and AGS‐EBV cells were seeded into a 6‐well plate and incubated for 24 h. Then, the lentiviruses were added to the wells. After 48 h, the tumor cells were selected with the help of puromycin (MP, 021 945 3925) to obtain stably transduced cells. These cells were cultured in complete RPMI 1640 medium (GIBCO, 10% FBS, NEZERUM) supplemented with puromycin and used for RNA extraction and supernatants collection.

### Clinical Analysis

Paraffin‐embedded sections were first deparaffinized and rehydrated. Antigen retrieval was performed with a citrate solution (pH 6.0) or Tris‐EDTA (pH 9.0) under high‐temperature and high‐pressure conditions. Endogenous peroxidase activity was blocked with 3% H_2_O_2_.

For immunohistochemical staining, the sections were then blocked with sheep serum for 1 h (ZS bio.co., ZLI‐9056), incubated with the primary antibody for 1 h and then incubated with the secondary antibody (Dako, GK500710) for half an hour at 37 °C. Afterward, DAB (ZS bio.co., ZLI‐9017) was used to stain the antibodies, and hematoxylin was used to stain the nuclei. The primary antibodies used were as follows: MPO (Abcam, ab9535, 1:100), CD66b (S‐766‐55, 1:2000), CD8 (ZA‐0508‐6.0), and CXCL10 (R&D, AF‐266‐NA, 1:1000).

For immunofluorescence staining, the sections were then blocked with donkey serum for 30 min (Absin, abs935) at room temperature. The sections were subsequently incubated with rabbit anti‐H3Cit (Abcam, ab5103, 1:100), goat anti‐MPO (R&D, AF3667, 1 µg mL^−1^), mouse anti‐CD8 (Abcam, ab17147, 1:200) or rabbit anti‐granzyme B (CST, 46890S, 1:200) antibodies overnight at 4 °C. The sections were incubated with Alexa Fluor‐conjugated secondary antibodies (Invitrogen) in 1% BSA for 1 h at room temperature. DAPI was used to counterstain the nuclei.

Multiplex immunohistochemical staining was conducted using a Panovue multiplex fluorescent IHC kit (0 004 100100, Panovue) according to the manufacturer's guidelines. The primary antibodies used were as follows: MPO (Abcam, ab9535, 1:50), CD8 (CST, 70 306, 1:100), Granzyme B (CST, 46890S, 1:50), and Pan‐CK (CST, 4545, 1:200).

All the tissues were scanned with KF‐PRO‐200, Olympus FV1000 or Olympus VS200 and analyzed using HALO or Qupath software. The samples were classified into high‐ or low‐expression groups based on the cutoff analyzed by the R programming language.

### Neutrophil Isolation

The peripheral blood from healthy volunteers was provided by the Knowcell Biotechnology Company. Human neutrophils were isolated from the peripheral blood via density gradient separation. The cell suspension was laid over 15 mL of Ficoll (LTS1077‐1) gradients in 50 mL tubes and centrifuged at 2500 rpm for 25 min at room temperature. Erythrocytes and neutrophils‐containing pellets were then mixed with red blood cell lysis buffer (C3702) and inverted for 30 min at 4 °C. After adequate mixing, the mixture was centrifuged at 1500 rpm for 5 min at 4 °C. The mixing process was repeated several times until the pellet was no longer red. The purity of the isolated neutrophils was determined by detecting CD15 and CD16 with flow cytometry. The neutrophil purity was > 95% (CD15‐positive and CD16‐positive neutrophils).

### NETs Formation Assays

For the SYTOX assay, 3 × 10^5 freshly isolated neutrophils from healthy donors were resuspended in the indicated supernatants and placed in black 96‐well plates (Corning, 3603) for incubation. SYTOX Green (5 nM, ThermoFisher) was added to each well, and CXCL10 (1–400 ng mL^−1^, PeproTech, 300‐12‐5) or AMG487 (1–4 µm, Selleck, S8682) was added, as indicated in the figures and figure legends. After 4 h, SYTOX Green fluorescence was measured with a Tecan Spark TM10M instrument.

For NETs visualization, 1 × 10^6 freshly isolated neutrophils were resuspended in 150 µL of different culture medium and placed in microscopy chambers (D35‐10‐1‐N). Stimuli, antibodies and inhibitors were added at the indicated concentrations. LPS (Sigma) 5 µg mL^−1^, DNase I (Roche) 0.5 U/well, CCL5 (MCE) 200 ng mL^−1^, CXCL10 (PeproTech) 200 ng mL^−1^, anti‐CXCL10 antibody (R&D) 1–4 µg mL^−1^, AMG487 (Selleck) 2 µm, GSK484 (MCE) 10 µm, Apocynin (MCE) 5 mm and N‐acetyl‐cysteine (Sigma) 5 mm. Neutrophils were incubated at 37 °C with 5% CO_2_ for 4 h, after which SYTOX Green (5 nM, ThermoFisher) was added for an additional 5‐min incubation period. The samples were then treated with anti‐Fade fluorescence mounting medium (Abcam, ab104135) and stored at 4 °C. To quantify NETs formation, Olympus FV1000 was utilized for scanning. The NETs area was quantified using ImageJ software. Only structures positive for SYTOX Green staining and depicting NETs morphology were selected for NETs quantification, and intact granulocyte nuclei were excluded from the analysis. NETs were counted in at least three fields per sample, and three samples were evaluated.

For immunocytochemistry staining, 1 × 10^6 freshly isolated neutrophils were resuspended in 150 µL of serum‐free medium supplemented with or without CXCL10 (200 ng mL^−1^, PeproTech, 300‐12‐5) and GSK484 (10 µm, MCE), and placed in microscopy chambers (D35‐10‐1‐N). Neutrophils were incubated at 37 °C with 5% CO2 for 4 h. Cells were fixed with 4% PFA for 30 min at room temperature, followed by 10 min of washing in PBS (three times), and 30 min of blocking with donkey serum (Absin, abs935) at room temperature. Cells were subsequently incubated with rabbit anti‐H3Cit (Abcam, ab5103, 1:100), and goat anti‐MPO (R&D, AF3667, 1 µg mL^−1^) antibodies for 60 min at room temperature. After rinsing twice with PBS, cells were incubated with Alexa Fluor‐conjugated secondary antibodies (Invitrogen) for 60 min at room temperature. DAPI was used to stain DNA for 15 min at room temperature. Cells were then treated with anti‐Fade fluorescence mounting medium (Abcam, ab104135) and stored at 4 °C. To quantify NETs formation, Olympus FV1000 was utilized for scanning. NETs were identified as the co‐expression of DNA, MPO and H3Cit, which was quantified by ImageJ software. NETs were counted in at least three fields per sample, and three samples were evaluated.

For immunocytochemistry staining of PBNs‐T cells‐tumor cells, 1 × 10^5 C666‐1 cells were resuspended in 150 µL of DMEM medium (GIBCO) (10% FBS, NEZERUM) and placed in microscopy chambers pre‐coated with Matrigel (Corning, 354 234). C666‐1 cells were cultured for 5 days and stained with cell tracker Orange CMRA Dye (Thermo Fisher, C2927) at 1 µm. 1 × 10^6 freshly isolated neutrophils were resuspended in 150 µL of serum‐free medium supplemented with or without CXCL10 (200 ng mL^−1^, PeproTech, 300‐12‐5) and placed in the chambers. The samples were incubated at 37 °C with 5% CO2 for 4 h, after which SYTOX Green (5 nM, ThermoFisher) was added for an additional 5‐min incubation period. 2 × 10^6 LPM2A‐TCR‐T cells were stained with Hoechst (Beyotime, C1028) and added to the chambers. After 20 min, the samples were treated with anti‐Fade fluorescence mounting medium and stored at 4 °C. Imaging was performed using a Nikon ECLIPSE Ti2 microscope.

For the video microscopy experiment, time‐lapse videos were captured with a Nikon CSU‐W1 microscope equipped with a cell culture chamber. A total of 1 × 10^5 freshly isolated neutrophils were resuspended in 10 µL of a mixture of 10% Matrigel (Corning, 354 234), 10% PureCol (Sigma, 5074‐35ML), and 80% different supernatants, as indicated in the figures or figure legends, and cultured in black 96‐well plates (Corning, 3603) for 40 min at 37 °C after the matrix solidified. Then, 100 µL of different supernatants containing 5 nM SYTOX Green (ThermoFisher) were added. Time‐lapse Z‐stackk videos of each well were captured every 10 min for 4 h. NIS‐Elements AR 5.30.01 software was used to analyze the fluorescence intensity.

### Chemotaxis Assay

A total of 5 × 10^5 freshly isolated neutrophils were resuspended in 200 µL of serum‐free medium and added to the upper 3 µm chamber (Corning, 353 096). The lower chamber was filled with 600 µL of serum‐free medium supplemented with varying concentrations of CXCL8 (3, 10, 30, 100, and 300 ng mL^−1^, PeproTech, 200–08M‐25), CXCL10 (3, 10, 30, 100, and 300 ng mL^−1^, PeproTech, 300‐12‐5) or CCL5 (3, 10, 30, 100, and 300 ng mL^−1^, PeproTech, 300‐06‐20). Neutrophils were incubated at 37 °C with 5% CO_2_ for 4 h. Triplicate wells of each condition were performed. Afterward, the upper chamber was carefully removed, and neutrophils that had migrated to the lower chamber were counted. The relative number of migrated neutrophils was calculated by normalization to serum‐free medium group.

A total of 5 × 10^5 freshly isolated neutrophils were treated with tumor cell supernatants supplemented with or without maraviroc (1 µm, MCE, HY‐13004) or AMG487 (1 µm, Selleck, S8682) overnight. The pan caspase inhibitor (3 µm, R&D, OPH001‐01 m) was added to inhibit spontaneous neutrophil apoptosis and increase its lifespan. Pretreated neutrophils were then resuspended in 200 µL of serum‐free medium and added to the upper 3 µm chamber (Corning, 353 096). The lower chamber was filled with 600 µL of tumor cell supernatants supplemented with or without CXCL10 (100 ng mL^−1^, PeproTech, 300‐12‐5) or CCL5 (100 ng mL^−1^, PeproTech, 300‐06‐20). Neutrophils were incubated at 37 °C with 5% CO_2_ for 4 h. Triplicate wells of each condition were performed. Afterward, the upper chamber was carefully removed, and neutrophils that had migrated to the lower chamber were counted. The relative number of migrated neutrophils were calculated by normalization to the supernatants of EBV^‐^ epithelial cancer cells.

### T Lymphocyte‐Mediated Cytotoxicity Assay

C666‐1‐A11‐LMP2A cells (1 × 10^5/well) were cultured in 48‐well plates overnight. Subsequently, 5 × 10^5 freshly isolated neutrophils were resuspended in tumor cell supernatants, as indicated in the figures, added to the plate and cultured for another 4 h at 37 °C with 5% CO_2_. DNase I (0.25 U/well, Roche, 10 104 159 001), CXCL10 (200 ng mL^−1^, PeproTech, 300‐12‐5) or AMG487 (2 µm, Selleck) was added to the system along with the neutrophils. Afterward, 5 × 10^5 LPM2A‐TCR‐T cells were added and incubated for an additional 16 h. The cells and supernatants were then collected separately. The cells were labeled with an Annexin V‐AF647/7‐AAD apoptosis kit (GOONIE, 100–103) and analyzed by flow cytometry. Neutrophils and LPM2A‐TCR‐T cells were excluded from the analysis by CD16^+^ and CD3^+^ staining, respectively.

AGS‐1‐A11‐LMP2A cells (3 × 10^5/well) were stained with cell tracker Orange CMRA Dye (1 µm, Thermo Fisher, C2927) and seeded in 24‐well plates overnight. The 1 × 10^6 freshly isolated neutrophils were added to the plate with or without CXCL10 (200 ng mL^−1^, PeproTech, 300‐12‐5) and incubated at 37 °C with 5% CO2 for 4 h, after which SYTOX Green (5 nM, ThermoFisher) was added for an additional 5‐min incubation period. The 5 × 10^6 LPM2A‐TCR‐T cells were stained with Hoechst (Beyotime, C1028) and added to the plate. After 16 h, imaging was performed using a Nikon ECLIPSE Ti2 microscope.

### Flow Cytometry

Cocultures of C666‐1‐A11‐LMP2A cells, neutrophils, and LPM2A‐TCR‐T cells were generated as described above. A cell activation cocktail (BioLegend, 423 304) was added to the coculture system 5 h before harvest. The cells were collected and stained with the Zombie UV Fixable Viability Kit (BioLegend, 423 107), anti‐human CD8 (BioLegend, 344 714), and tetramers (HLA‐A*11:01 EBV LMP2 tetramer‐SSCSSCPLSK‐PE, TS‐M111‐1) to identify LPM2A‐TCR‐T cells. After centrifugation and removal of the supernatant, the cells were fixed and permeabilized with a Fixation/Permeabilization Kit (BD, 554 714) to stain intracellular molecules. Briefly, the cells were treated with Cytofix/Cytoperm solution for 30 min and washed with Perm/Wash solution. Anti‐human granzyme B (BD, 563 389), anti‐human IFN‐γ (BioLegend, 502 512), and anti‐human TNF‐α (BioLegend, 502 909) antibodies were added at 1:100 dilutions for intracellular staining and incubated for 15 min on ice. Finally, the cells were resuspended in 300 µL of PBS and analyzed.

For examining CXCR3 expressed on neutrophils, 1 × 10^6 freshly isolated neutrophils were resuspended in 1 mL of different supernatants, as indicated in the figures and figure legends, and the cells were then added to a 24‐well plate and incubated at 37 °C with 5% CO_2_ for 4 h. Afterward, these cells were collected and stained with Fixable Viability Stain 780 (BD, 565 388), anti‐human CD15 (BioLegend, 301 904), and anti‐human CXCR3 (BioLegend, 353 720) antibodies. Finally, the cells were resuspended in 300 µL of PBS and analyzed.

### ELISA

Tumor cell supernatants were collected and analyzed for the presence of CCL5 or CXCL10 with a CCL5 ELISA kit (Proteintech, KE00093) or a CXCL10 ELISA kit (Neobioscience, EHC157.96), according to the manufacturers’ protocols. The supernatants from the cocultures of C666‐1‐A11‐LMP2A cells and LPM2A‐TCR‐T cells with or without neutrophils were collected and analyzed for perforin or tumor necrosis factor alpha (TNF‐α) levels with a perforin ELISA kit (Neobioscience, EHC154.96) or a TNF‐α ELISA kit (FineTest, EH0302), according to the manufacturers’ protocols.

### RNA Extraction and Quantitative Real‐Time PCR

Total RNA was extracted from tumor cells using an NcmSpin Cell/Tissue Total RNA Kit (NCM, M5105), according to the manufacturer's instructions. The concentration and purity of the RNA were determined with a Nanodrop 2000 spectrophotometer (ThermoFisher Scientific). The RNA from each sample was reverse transcribed using the GoScript Reverse Transcription System (Promega, Madison, WI, USA) and then used for quantitative real‐time PCR with the GoTaq qPCR Master Mix Kit (Promega, Madison, WI, USA). The sequences of primers used were as follows: CCL5 forward, 5′‐TTCTACACCAGTGGCAAGTG‐3′, reverse, 5′‐CCATCCTAGCTCATCTCCAAAG‐3′; CXCL10 forward, 5′‐TCCACGTGTTGAGATCATTGC‐3′, reverse, 5′‐TCTTGATGGCCTTCGATTCTG‐3′; and GAPDH forward, 5′‐GCATTGCCCTCAACGACCAC‐3′, reverse, 5′‐CCACCACCCTGTTGCTGTAG‐3′. GAPDH was used as an endogenous control for normalization.

### Transcriptome Analysis

The global gene expression profiles of EBV^−^ and EBV^+^ epithelial cancer cells were subjected to RNA‐seq analysis, as previously described.^[^ ^59^ ^]^ Gene Ontology (GO) functional classification of the differentially expressed genes (DEGs) was conducted using DAVID (https://david.ncifcrf.gov/). Cytoscape software 3.7.2 with the cytoHubba and ClueGO plug‐ins was used to visualize the PPI networks of these DEGs and screen core genes and biological processes.^[^ ^19^ ^]^

### Animal Studies

For NETs detection in the C666‐1‐A11‐LMP2A tumor model, 4‐week‐old female NCG mice were injected with 5 × 10^6 C666‐1‐A11‐LMP2A cells in the right flanks. When the tumor volume reached ≈50 mm^3^, the mice were injected with neutrophils. Before the injection, neutrophils from peripheral blood were stained with CELLTRACKER ORANGE CM (ThermoFisher, C2927) and Hoechst (ThermoFisher, 62 249) for 20 min at 37 °C and washed three times. A total of 1 × 10^7 neutrophils were injected into mice with or without DNase I (Roche, 50 U/mouse) via the tail vein. Twenty‐four hours later, each mouse was i.v. injected with 50 µL of SYTOX Deep Red (10 µm, Invitrogen, S11380), and 5 min later, the mice were sacrificed. Tumors were collected and cut into frozen sections, which were then scanned with a KF‐PRO‐200 and analyzed using HALO software. NETs were characterized by the co‐expression of CELLTRACKER ORANGE CM, Hoechst and SYTOX Deep Red.

For the C666‐1‐A11‐LMP2A tumor growth studies, 5 × 10^6 C666‐1‐A11‐LMP2A cells were subcutaneously injected into the right flanks of 4‐week‐old female NCG mice. When the tumor volume reached ≈50 mm^3^, 1 × 10^7 freshly isolated neutrophils from healthy donors were injected into the mice with or without DNase I. One week later, 1 × 10^7 LPM2A‐TCR‐T cells were injected into the mice via the tail vein along with IL‐2 (10 000 IU). IL‐2 was injected intraperitoneally at 2‐day intervals. Tumors were measured every two days with calipers, and the tumor volume was calculated according to the following equation: small diameter^2^ × large diameter × 0.5. The mice were sacrificed the day after the last injection of LPM2A‐TCR‐T cells, and the xenograft tumors were harvested for further analyses.

All NCG mice were obtained from Gempharmatech Company, and the animal experiments were approved by the Institutional Animal Care and Use Committee of Sun Yat‐sen University (Ethical Approval No. L102042020120E).

### Statistical Analysis

GraphPad Prism 9 and SPSS Statistics 25.0 were used for statistical analyses. The statistical methods used are indicated in the figure legends. Significance was determined as ^*^ *p* < 0.05, ^**^ *p* < 0.01, ^***^ *p* < 0.001, and ^****^ *p* < 0.0001, n.s., not significant. The in vitro experiments were repeated independently multiple times, as indicated in the figure legends. SPSS Statistics 25.0 was used to analyze the clinical information of the patients.

## Conflict of Interest

The authors declare no conflict of interest.

## Author Contributions

D.O., T.X., and Y.C. contributed equally to this work. D.O. designed the study, conducted most of the experiments and wrote the article. T.X. provided financial support, participated in some of the experiments, and helped writing the article. Y.C. participated in many of the experiments. M.S. provided financial support and technical guidance. J.Z. and D.W. were helpful for providing technical guidance for some experiments. H.C. helped with animal studies. S.L. and L.Z. helped with constructing C666‐1‐A11‐LPM2A cells and LPM2A‐TCR‐T cells. C.X., Y.R., Y.T., and S.X. helped with collecting blood samples from healthy donors. Q.W., J.H., Y.L., and J.Y. helped with flow cytometry experiments. Y.L., Y.W., and X.Y. helped with clinical analysis. S.X. and Y.L. helped with isolating neutrophils. Q.P. and Q.Y. were responsible for designing the study, providing theoretical guidance and revising the article. J.X. was responsible for designing the study, providing financial and theoretical guidance, and revising the article.

## Supporting information



Supporting Information

Supplemental Video 1

Supplemental Video 2

Supplemental Video 3

Supplemental Video 4

Supplemental Video 5

Supplemental Video 6

Supplemental Video 7

Supplemental Video 8

## Data Availability

The authors declare that the main data supporting the findings of this study are available within the article and its supporting information files. Extra data are available from the corresponding authors upon reasonable request.
