# Supplementary material for: The CXCL10‐CXCR3 Axis Induces Tumor‐Associated Neutrophils to Interfere with CTLs‐Mediated Antitumor Activity in EBV‐Associated Epithelial Cancers
Source: Adv Sci (Weinh). 2025 Jul 21;12(39):e00950. doi: 10.1002/advs.202500950 (PMC12533379; doi:10.1002/advs.202500950)
Supplement: Supplementary file 1 — Supporting Information [file ADVS-12-e00950-s009.docx]

**The CXCL10-CXCR3 Axis Induces Tumor-Associated Neutrophils to Interfere with CTLs-Mediated Antitumor Activity in EBV-Associated Epithelial Cancers**

Dijun Ouyang^1, 2#^, Tong Xiang^1, 3#^, Yuanyuan Chen^1, 2#^, Mengjia Song^1, 4^, Jingjing Zhao^1, 2^, Hao Chen^1, 2^, Si Li^5^, Lifeng Zhang^5^, Chi Xu^6^, Yan Ren^6^, Yong Tao^6^, Qijing Wang^1, 2^, Jia He^1, 2^, Yongqiang Li^1, 2^, Sisi Xie^6^, Yuanyuan Liu^1, 2^, Yan Wang^7^, Xinyi Yang^1, 2^, Jinqi You^1^, Songzuo Xie^1, 8^, Yingzi Li^1, 2^, Desheng Weng^1, 2^, Qiuzhong Pan^1, 2*^, Qi Yang^1, 9*^, Jianchuan Xia^1, 2*^

1 State Key Laboratory of Oncology in South China, Guangdong Provincial Clinical Research Center for Cancer, Guangdong Key Laboratory of Nasopharyngeal Carcinoma Diagnosis and Therapy, Sun Yat-sen University Cancer Center, Guangzhou, Guangdong, 510060, P.R. China.

2 Department of Biotherapy, Sun Yat-sen University Cancer Center, Guangzhou, Guangdong, 510060, P.R. China.

3 Department of Experimental Research, Sun Yat-sen University Cancer Center, Guangzhou, Guangdong, 510060, P. R. China.

4 Department of Pediatric Oncology, Sun Yat-sen University Cancer Center, Guangzhou, Guangdong, 510060, P.R. China.

5 TCRCure Biological Technology Co., Ltd, Guangzhou, China.

6 Knowcell Biotechnology Co., Ltd, Shenzhen, China.

7 Department of Medical Oncology, The Third Affiliated Hospital of Sun Yat-sen University, 600 Tianhe Road, Guangzhou 510630, China.

8 Department of Nuclear Medicine, Sun Yat-sen University Cancer Center, Guangzhou 510060, P. R. China.

9 Department of Nasopharyngeal Carcinoma, Sun Yat-sen University Cancer Center, Guangzhou 510060, P. R. China.

^#^Dijun Ouyang, Tong Xiang and Yuanyuan Chen have contributed equally to this study.

^*^Corresponding authors:

Jianchuan Xia, Email: xiajch@mail.sysu.edu.cn

Qi Yang, Email: yangqi@sysucc.org.cn

Qiuzhong Pan, Email: panqzh@sysucc.org.cn.

**
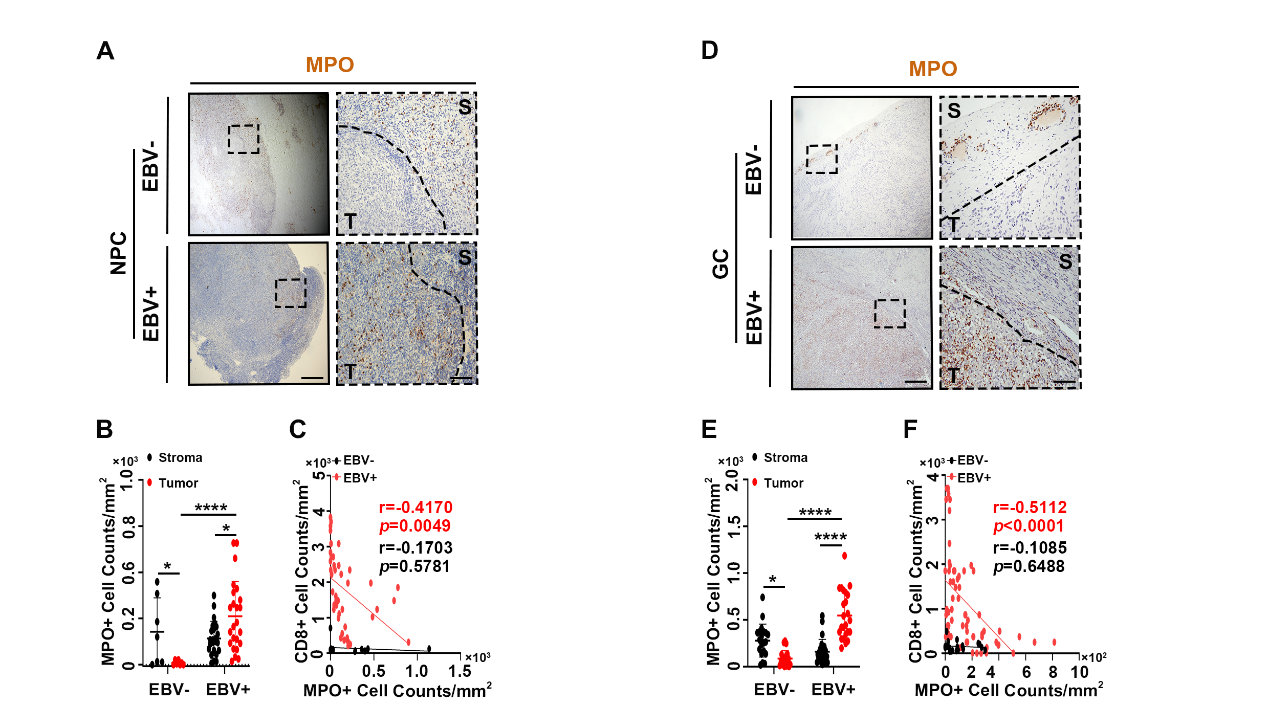
**

**Figure S1.**

MPO^+^ cells are elevated and negatively correlated with CD8^+^ cells in EBV-associated epithelial cancers. A,D) Representative images of serial sections from EBV^-^ and EBV^+^ NPC (A) and GC (D) stained for MPO. Magnifications: 40x (black solid line frame), 200x (black dashed line frame). “S” indicates the stroma area, and “T” indicates the tumor area. Scale bars: 500 μm (40x images), 100 μm (200x images). B,E) Statistical analysis of the density of MPO^+^ cells in NPC (B) and GC (E). C,F) Correlation analysis between MPO^+^ cells and CD8^+^ cells in NPC (C) and GC (F). NPC: nasopharyngeal cancer. GC: gastric cancer. EBER: Epstein-Barr virus-encoded small RNA. Mean ± SD are shown for all panels including error bars. *p* values were calculated with Mann-Whitney *U* test or two-tailed *t*-test. **p* < 0.05, ***p* < 0.01, ****p* < 0.001, and *****p* < 0.0001, n.s., not significant.


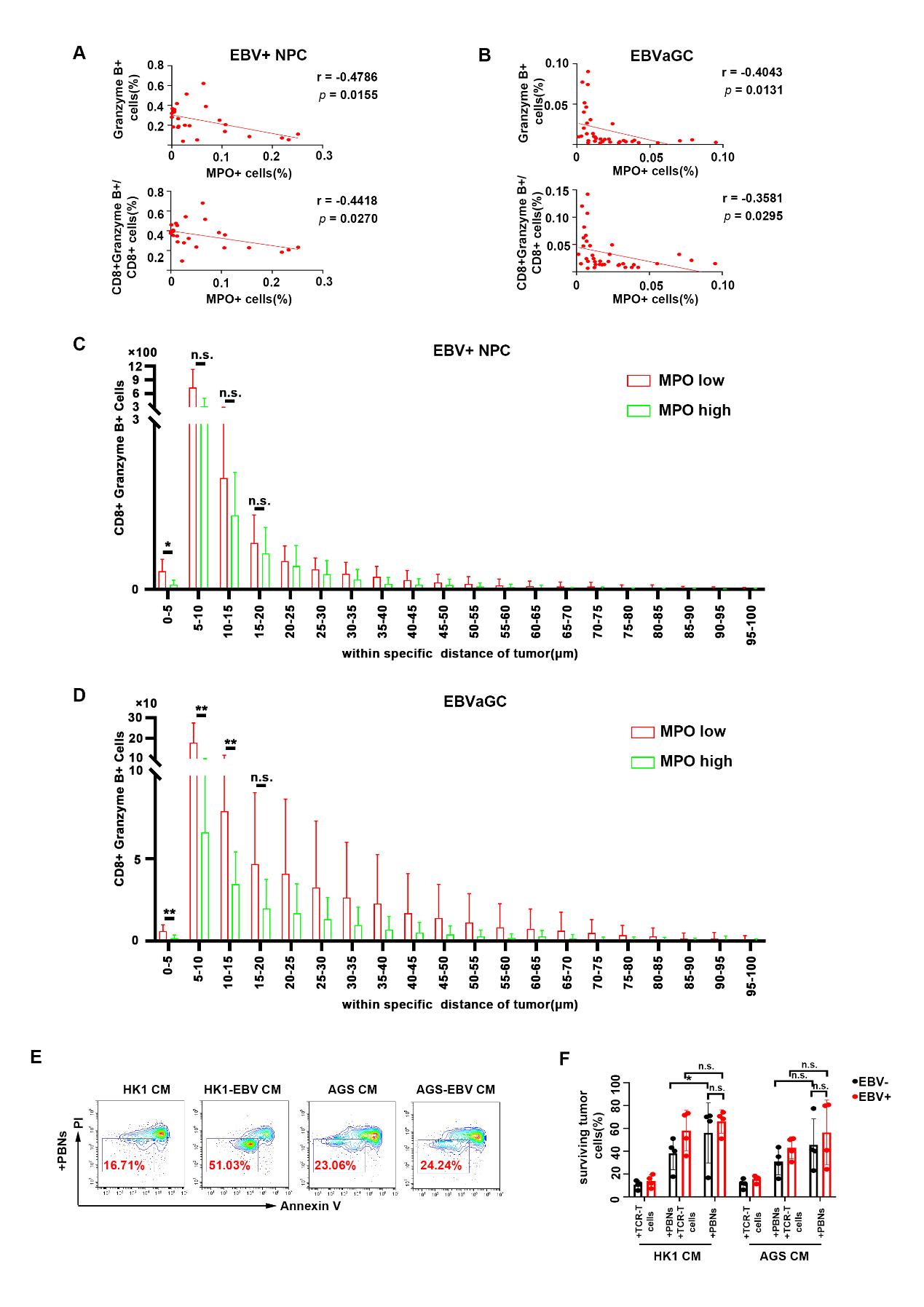


**Figure S2.**

TANs in EBV-associated epithelial cancers are negatively correlated with the cytotoxicity of CTLs. A,B) Correlation analysis between MPO^+^ cells and granzyme B^+^ cells (upper panel) or the ratio of CD8^+^ granzyme B^+^/ CD8^+^ cells (lower panel) in EBV^+^ NPC (A) and EBVaGC (B), as determined by multiplex immunohistochemistry. two-tailed *t*-test. *n* = 3. C,D) Proximity analysis was conducted to analyze the number of CD8⁺ granzyme B⁺ cells at specific distances within 100 µm from the tumor cells in regions with high or low expression of MPO in EBV^+^ NPC (C) and EBVaGC (D), as determined by multiplex immunohistochemistry. Mean ± SD, one-way ANOVA, *n* = 3. E,F) Representative images (E) and analysis (F) of surviving C666-1-A11-LMP2A cells cocultured with neutrophils freshly isolated from healthy donors in the supernatants of EBV^-^ and EBV^+^ epithelial cancer cells for 20 h. Each dot represents an individual repetition of the experiment with neutrophils from different donors, and results were compared using a ratio paired *t*-test. *n* = 4 donors. NPC: nasopharyngeal cancer. EBVaGC: EBV-associated gastric cancer. PBNs: peripheral blood neutrophils. **p* < 0.05, ***p* < 0.01, ****p* < 0.001, and *****p* < 0.0001, n.s., not significant.

**
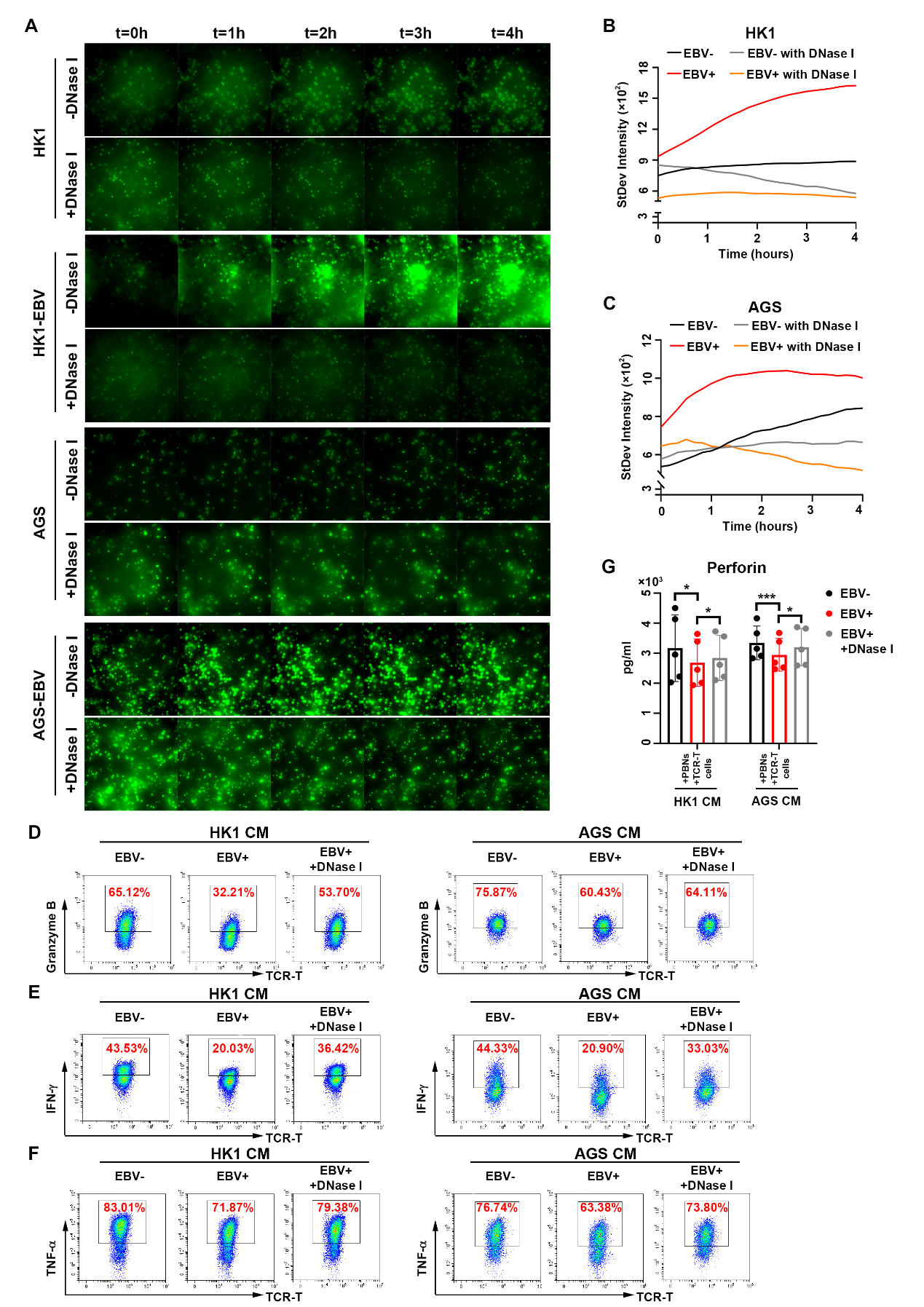
**

**Figure S3.**

DNase I can digest NETs and therefore rescue T lymphocyte-mediated cytotoxicity. A-C) Time-lapse videos of NETs formation from freshly isolated neutrophils cultured in the supernatants of EBV^-^ and EBV^+^ epithelial cancer cells for 4 h, with the presence of SYTOX Green (green) and with or without DNase I (0.5 U). Representative frames of videos at different time points (A) and quantification of NETs extrusion in supernatants of HK1 cells (B) or AGS cells (C). D-F) C666-1-A11-LMP2A cells were cocultured with neutrophils freshly isolated from healthy donors in the supernatants of EBV^-^ and EBV^+^ epithelial cancer cells for 4 h supplemented with or without DNase I (0.5 U). LPM2A-TCR-T cells were then added and collected after 16 hours for testing the expression of granzyme B, IFN-γ and TNF-α. Representative images showing the expression of granzyme B (D), IFN-γ (E) and TNF-α (F) tested by flow cytometry. G) Supernatants obtained from the experiment as in (D) were analyzed for perforin expression using ELISA. Mean ± SD. ratio paired *t*-test. *n* = 5 donors. TCR-T cells: LPM2A-TCR-T cells. PBNs: peripheral blood neutrophils. **p* < 0.05, ***p* < 0.01, ****p* < 0.001, and *****p* < 0.0001, n.s., not significant.


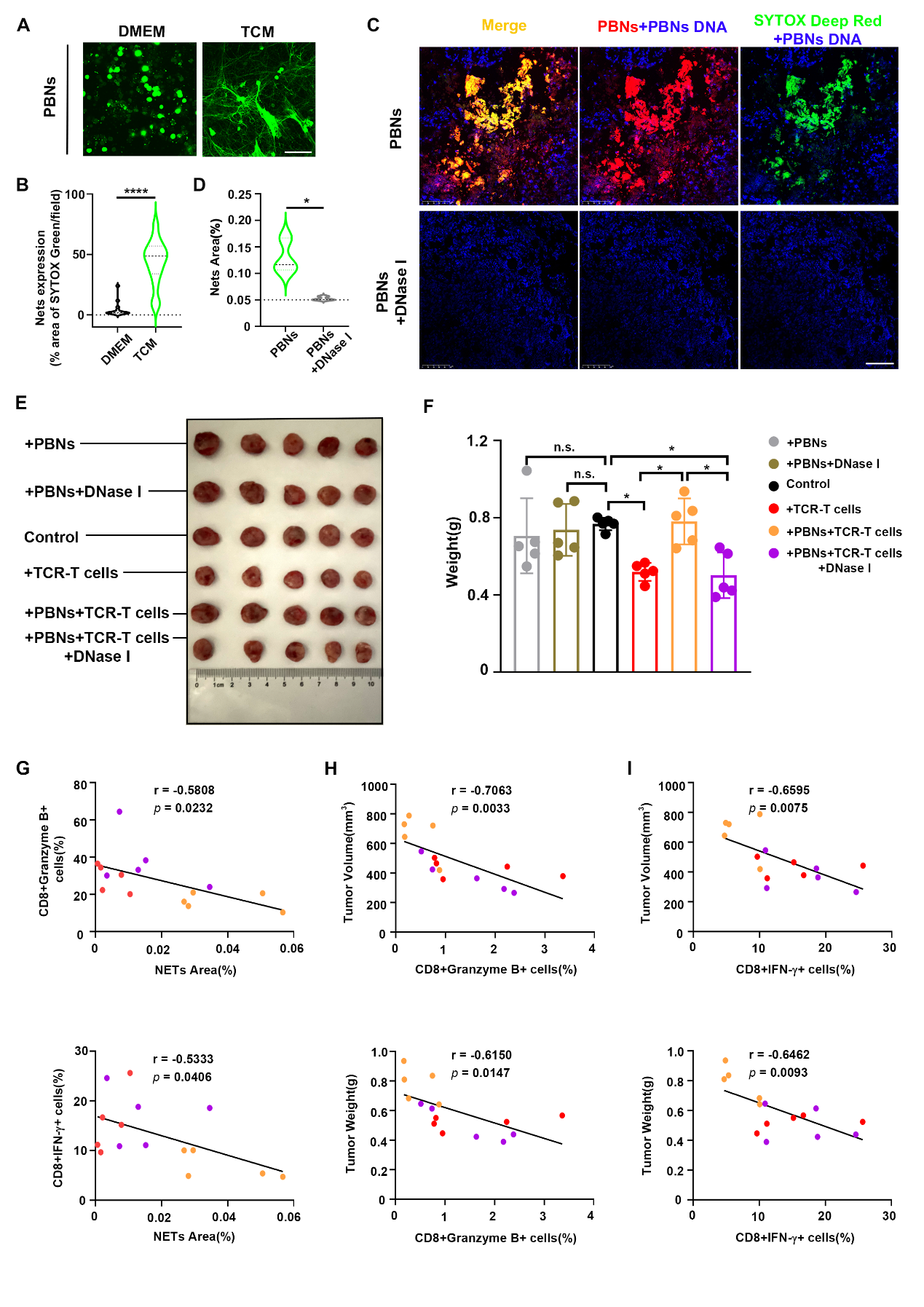
**Figure S4.**

NETs formed *in vivo* suppress T lymphocyte-mediated antitumor activity. A) Neutrophils freshly isolated from healthy donors were cultured in the supernatant of C666-1-A11-LMP2A cells or serum-free DMEM for 4 h and stained with SYTOX Green (green) to evaluate NETs. Only structures positive for SYTOX Green staining and depicting NETs morphology were identified as NETs. Magnification: 400x Oil. Scale bar, 50 µm. B) Statistical analysis of NETs expression in (A). Mann-Whitney *U* test. *n* = 3. C) NCG mice planted with C666-1-A11-LMP2A cells were i.v. injected with neutrophils pre-stained with CELLTRACKER ORANGE CM (red) and Hoechst (blue), with or without DNase I (50 U/mouse). 24 hours later, mice were i.v. injected with SYTOX Deep Red (green, 10 µM/mouse) and were sacrificed 5 minutes later. NETs were defined as the co-expression of CELLTRACKER ORANGE CM, Hoechst and SYTOX Deep Red. Magnification: 200x. Scale bar, 100 µm. D) Statistical analysis of NETs expression in (C). Mann-Whitney *U* test. *n* = 3. E) Images of C666-1-A11-LMP2A xenograft tumors in NCG mice with different treatment as indicated. F) Statistical analysis of the weight of C666-1-A11-LMP2A xenograft tumors from (E). *n* = 5. G) Correlation analysis between NETs and CD8^+^ granzyme B^+^ T cells (up panel) or CD8^+^ IFN-γ^+^ T cells (lower panel) detected by flow cytometry. Red, orange and purple dots represent tumor sections from mice injected with LPM2A-TCR-T cells, LPM2A-TCR-T cells plus neutrophils, and LPM2A-TCR-T cells plus neutrophils plus DNase I, respectively. *n* = 15. H) Correlation analysis between CD8^+^ granzyme B^+^ T cells detected by immunofluorescence staining and the C666-1-A11-LMP2A xenograft tumor volume (up panel) or the C666-1-A11-LMP2A xenograft tumor weight (lower panel). Red, orange and purple dots represent tumor sections from mice injected with LPM2A-TCR-T cells, LPM2A-TCR-T cells plus neutrophils, and LPM2A-TCR-T cells plus neutrophils plus DNase I, respectively. *n* = 15. I) Correlation analysis between CD8^+^ IFN-γ^+^ T cells detected by flow cytometry and the C666-1-A11-LMP2A xenograft tumor volume (up panel) or the C666-1-A11-LMP2A xenograft tumor weight (lower panel). Red, orange and purple dots represent tumor sections from mice injected with LPM2A-TCR-T cells, LPM2A-TCR-T cells plus neutrophils, and LPM2A-TCR-T cells plus neutrophils plus DNase I, respectively. *n* = 15. TCM: tumor culture medium from C666-1-A11-LMP2A cells. TCR-T cells: LPM2A-TCR-T cells. PBNs: peripheral blood neutrophils. Mean ± SD are shown for all panels including error bars. *p* values were calculated with Mann-Whitney *U* test or two-tailed *t*-test. **p* < 0.05, ***p* < 0.01, ****p* < 0.001, and *****p* < 0.0001, n.s., not significant.

**
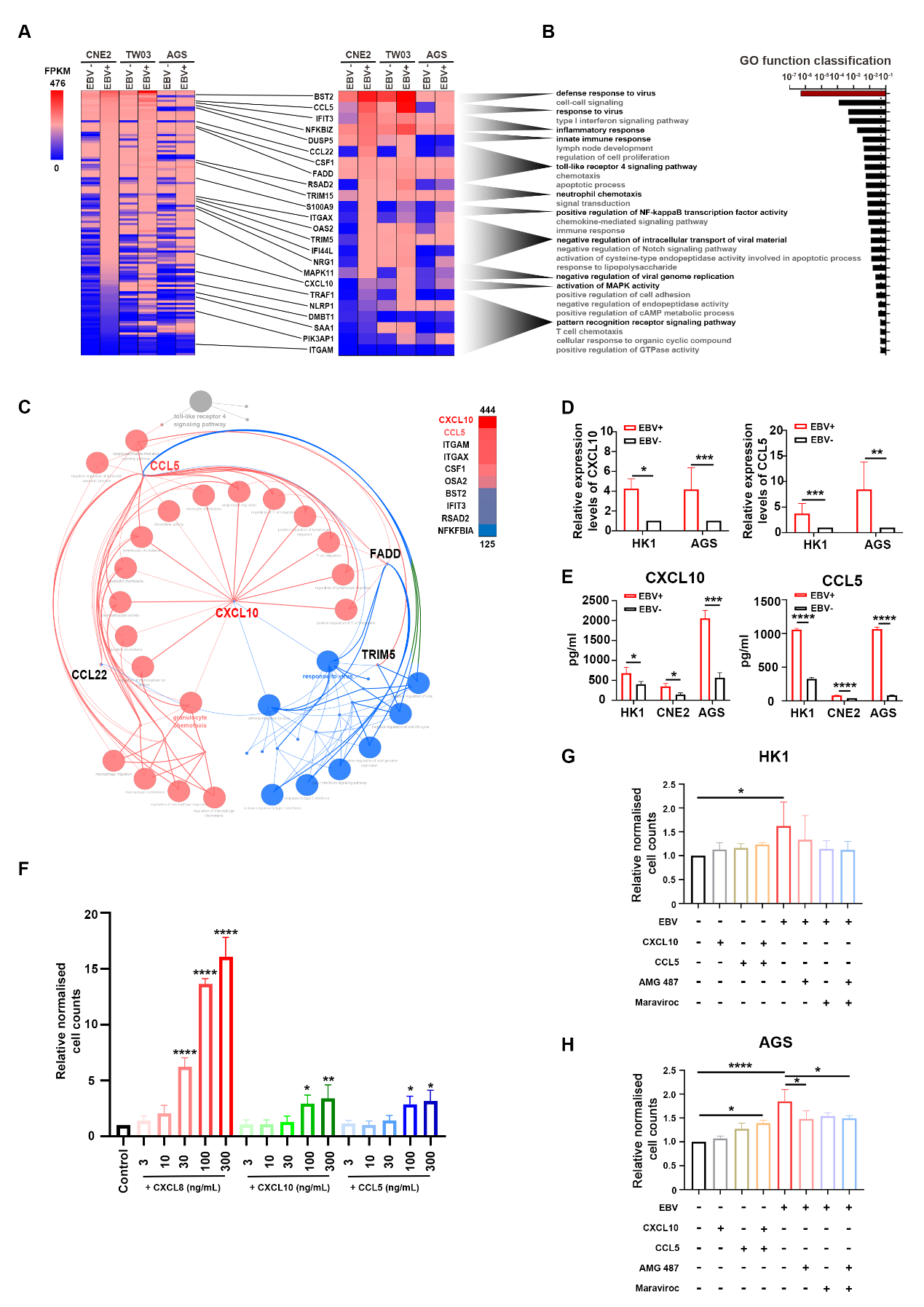
**

**Figure S5.**

CXCL10 increases in EBV-associated cancer cells and is involved in neutrophils chemotaxis. A) Heatmap showing the upregulated differentially expressed genes (DEGs) between EBV^-^ and EBV^+^ epithelial cancer cell lines (left panel) and those genes involved in response to virus, inflammatory response and NETs formation pathways (right panel). B) Bar plot ranking of the top enrichment score (*p*-value) values for the significant enrichment according to Gene Ontology (GO) function classification of DEGs in (A). Only enrichment with *p* < 0.05 are shown. Pathways related to response to virus, inflammatory response and NETs formation were marked as black. C) Functionally grouped network of biological process of the DEGs in (A) was visualized by Cytoscape with the cytoHubba and the ClueGo plug-in. The top 10 hub genes were selected and the two genes involved in response to virus and granulocyte chemotaxis, CXCL10 (red) and CCL5 (pink), were marked. D,E) CXCL10 and CCL5 expressed by EBV^-^ and EBV^+^ epithelial cancer cells were detected by quantitative real-time PCR (D) and ELISA (E). Mean ± SD. Mann-Whitney *U* tests or two-tailed *t*-test. F) Freshly isolated neutrophils were suspended in serum-free medium and placed to the 3 µm upper chamber and the different medium were placed into the lower chamber as indicated in the figure. After 4 hours, the recruited neutrophils in lower chamber were collected and counted. Mean ± SD. one-way ANOVA. G,H) Freshly isolated neutrophils were pre-treated overnight with culture supernatant from HK1 or HK1-EBV cells (G), AGS or AGS-EBV cells (H), supplemented with AMG487 (1 µM) or Maraviroc (1 µM). The pre-treated cells were then suspended in serum-free medium and placed to the 3 µm upper chamber and the supernatants of tumor cells were placed into the lower chamber, with CCL5 (100 ng mL^-1^) and/or CXCL10 (100 ng mL^-1^) added. After 4 hours, the recruited neutrophils in lower chamber were collected and counted. Mean ± SD. one-way ANOVA. **p* < 0.05, ***p* < 0.01, ****p* < 0.001, and *****p* < 0.0001, n.s., not significant.


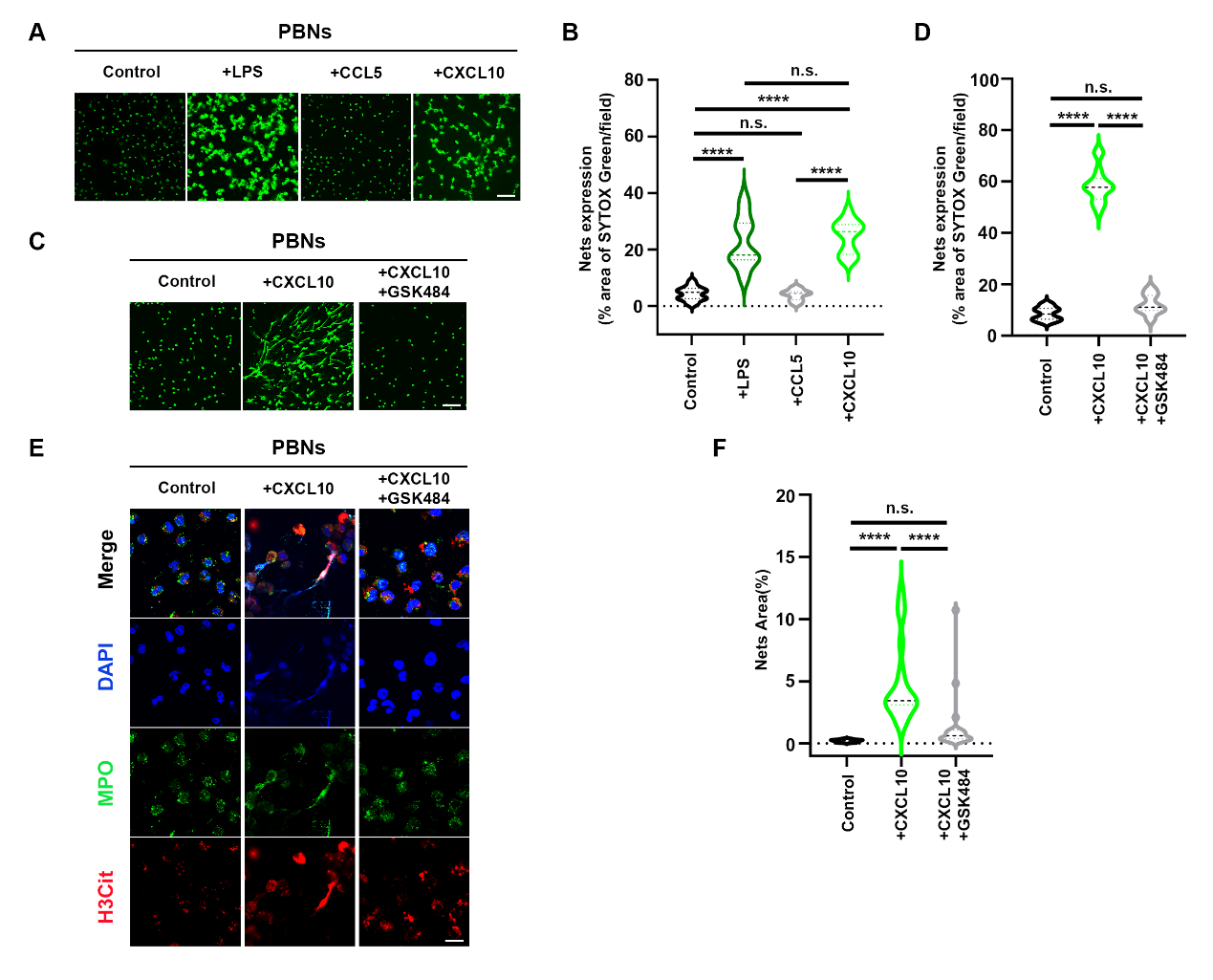


**Figure S6.**

CXCL10 induces NETs formation. A) Neutrophils freshly isolated from healthy donors were cultured in RPMI 1640 with or without LPS (5 µg ml^-1^), CCL5 (200 ng ml^-1^) or CXCL10 (200 ng ml^-1^) for 4 h, and stained with SYTOX Green (green) to evaluate Nets. B) Quantification of NETs was verified on base of 3 different donors. Magnification: 400x Oil. Scale bar, 50 µm. Mann-Whitney *U* test. C,E) Neutrophils freshly isolated from healthy donors were cultured in RPMI 1640 with or without CXCL10 (200 ng ml^-1^) and GSK484 (10 µM) for 4 h. (C) shows the representative images when NETs were visualized by staining with SYTOX Green (green). Magnification: 400x Oil. Scale bar, 50 µm. (E) shows the representative merges or single-stained images when NETs were visualized by staining with DAPI (blue), MPO (green) and H3Cit (red). NETs were identified by the co-expression of DNA (DAPI), MPO and H3Cit. Magnification: 1000x Oil. Scale bar, 20 µm. D,F) Quantification of NETs. (D) shows the statistical analysis of NETs expression in (C), and (F) shows the statistical analysis of NETs expression in (E). Mann-Whitney *U* test. *n* = 3. PBNs: peripheral blood neutrophils. **p* < 0.05, ***p* < 0.01, ****p* < 0.001, and *****p* < 0.0001, n.s., not significant.


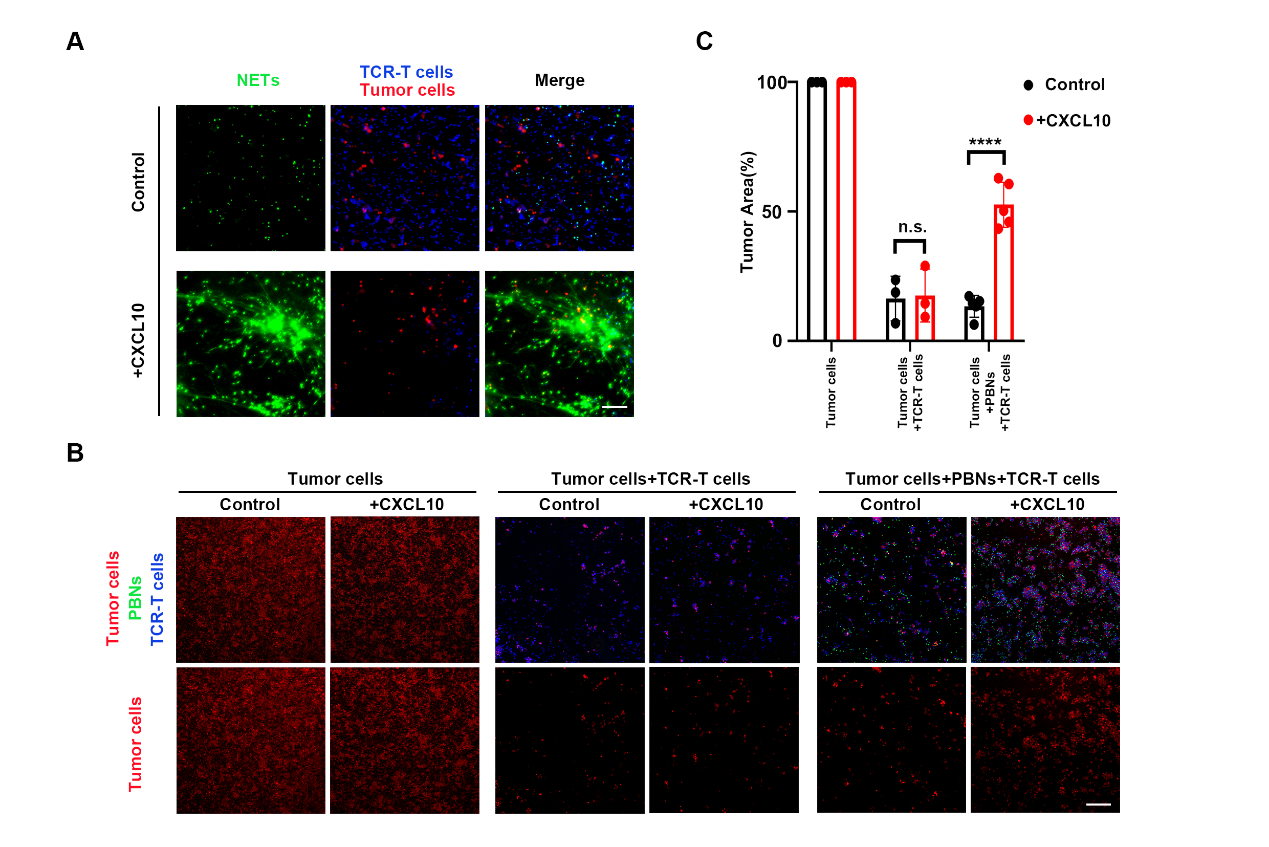


**Figure S7.**

CXCL10-induced NETs interfere with TCR-T cells cytotoxicity. A) Representative single-stained and merged images of tumor cells (red) cocultured with PBNs and LPM2A-TCR-T cells (blue) in the absence or presence of CXCL10 (200 ng ml^-1^). NETs were stained by SYTOX Green (green). Magnification: 100x. Scale bar, 200 µm. B) Representative images of surviving tumor cells (red) after cocultured with PBNs (green) for 4 h and LPM2A-TCR-T cells (blue) for additional 16 h in the absence or presence of CXCL10 (200 ng ml^-1^). Magnification: 40x. Scale bar, 500 µm. C) Quantification of the area occupied by the surviving tumor cells. Mean ± SD. two-tailed *t*-test. *n* = 5. TCR-T cells: LPM2A-TCR-T cells. PBNs: peripheral blood neutrophils. **p* < 0.05, ***p* < 0.01, ****p* < 0.001, and *****p* < 0.0001, n.s., not significant.

**
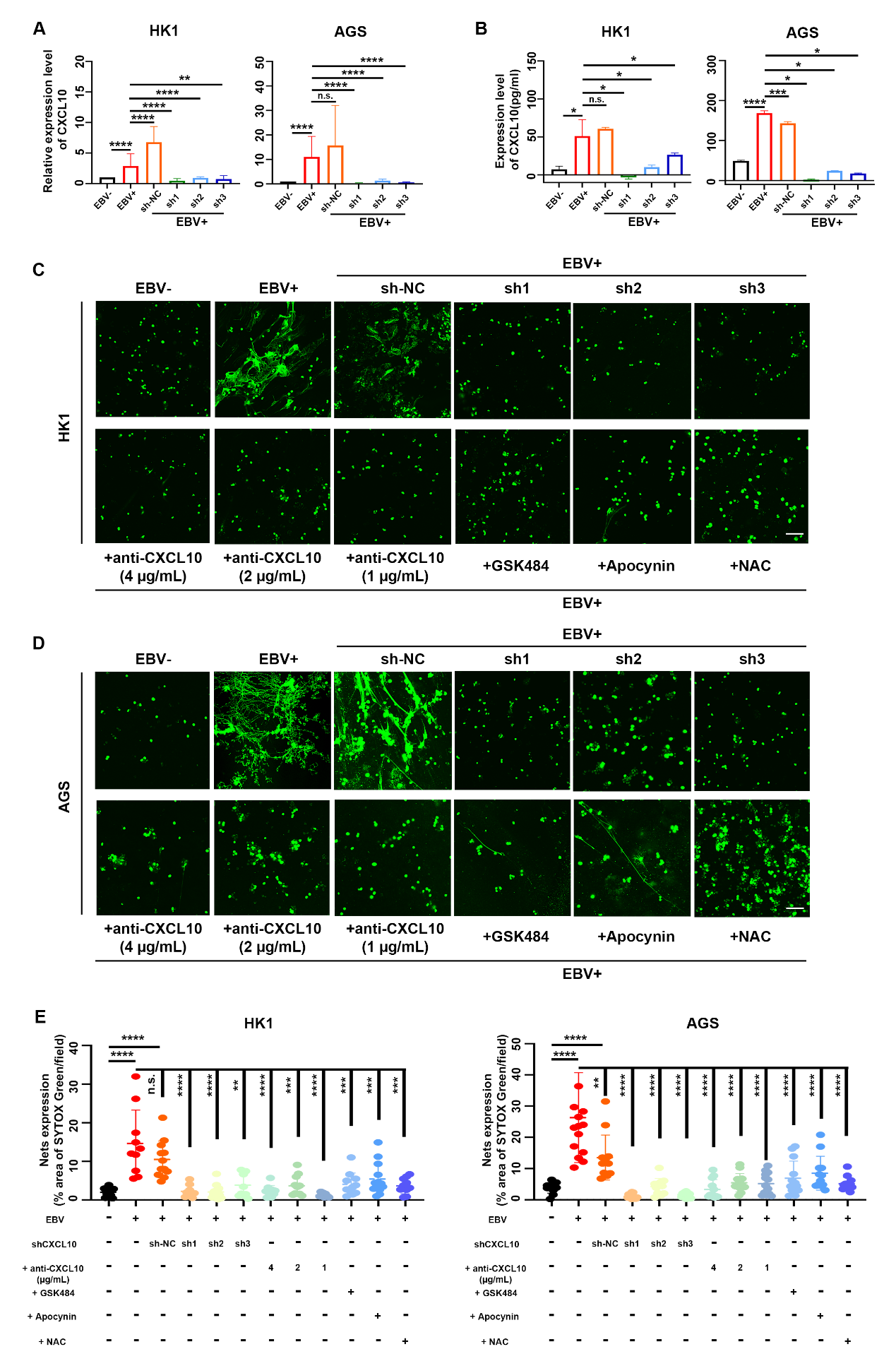
**

**Figure S8.**

CXCL10, ROS and PAD4 are involved in NETs formation induced by EBV-associated cancer cells. A,B) CXCL10 were knockdown in HK1-EBV and AGS-EBV cells and the expression of CXCL10 was verified by quantitative real-time PCR (A) and ELISA (B). C,D) Freshly isolated neutrophils were cultured in supernatants of HK1 cells, HK1-EBV-shCXCL10 cells or HK1-EBV cells supplemented with or without anti-CXCL10 antibody (1-4 µg ml^-1^), GSK484 (10 µM), Apocynin (5 mM) or N-acetyl-cysteine (5 mM) for 4 h, and stained with SYTOX Green (green) to test NETs formation (C). This experiment was repeated using the supernatants of AGS cells, AGS-EBV-shCXCL10 cells or AGS-EBV cells (D). Magnification: 400x Oil. Scale bar: 50 µm. E) Quantification of NETs formed in (C) and (D). *n* = 3. Mean ± SD are shown for all panels including error bars. *p* values were calculated with Mann-Whitney *U* test or two-tailed *t*-test. **p* < 0.05, ***p* < 0.01, ****p* < 0.001, and *****p* < 0.0001, n.s., not significant.


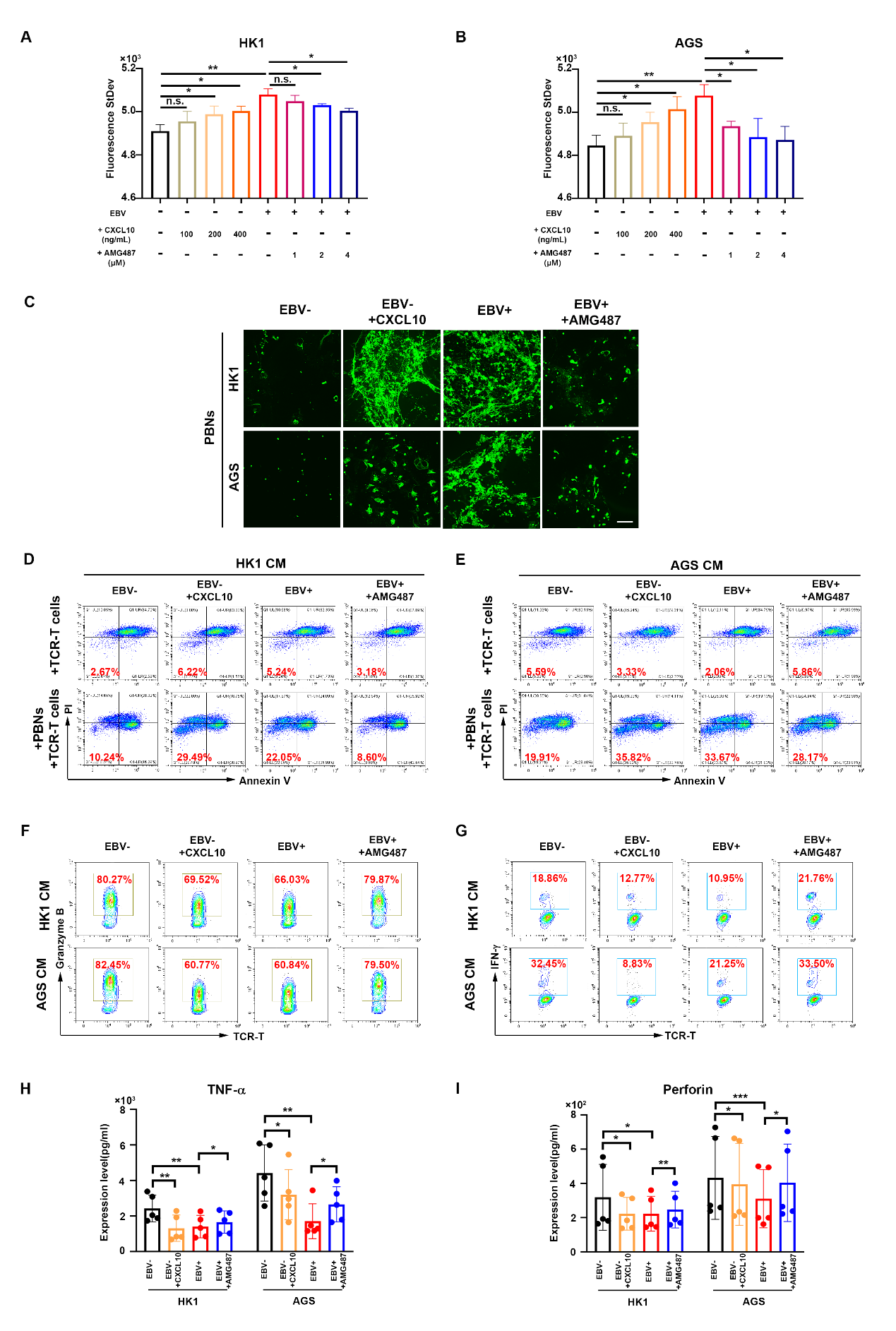


**Figure S9.**

EBV-mediated CXCL10-CXCR3 axis facilitates NETs formation. A,B) 3×10^5 neutrophils were suspended in supernatants of EBV^-^ or EBV^+^ HK1 (A), EBV^-^ or EBV^+^ AGS (B) cells supplemented with or without CXCL10 (1-400 ng ml^-1^) or AMG487 (1-4 µM), and placed into black 96-well plate for SYTOX assay. After 4 hours, SYTOX Green fluorescence was measured to determine NETs formation. C) 1×10^6 freshly isolated neutrophils were cultured in the supernatants of EBV^-^ or EBV^+^ epithelial cancer cells supplemented with or without CXCL10 (200 ng ml^-1^) or AMG487 (2 µM) for 4 hours, and SYTOX Green (green) was added to mark NETs formation. Magnification: 400x Oil. Scale bar: 50 µm. D,E) C666-1-A11-LMP2A cells were cocultured with neutrophils for 4 h and LPM2A-TCR-T cells for another 16 h in the indicated supernatants. Representative images of surviving C666-1-A11-LMP2A cells tested by flow cytometry. F,G) Representative images of granzyme B (F) and IFN-γ (G) expressed by LPM2A-TCR-T cells from the coculture system in (D) and (E). H,I) The expression of TNF-α (H) and perforin (I) in culture supernatants collected from the coculture system in (D) and (E) were examined by ELISA. Each dot represents an individual repetition of the experiment with neutrophils from different donors, and the results were compared using a ratio paired *t*-test. *n* = 5 donors. TCR-T cells: LPM2A-TCR-T cells. PBNs: peripheral blood neutrophils. Mean ± SD are shown for all panels including error bars. *p* values were calculated with Mann-Whitney *U* test or two-tailed *t*-test unless indicated. **p* < 0.05, ***p* < 0.01, ****p* < 0.001, and *****p* < 0.0001, n.s., not significant.

**Table S1. Association of NETs expression with clinicopathological features in patients with** **EBV^+^ NPC.**

|  |  | **EBV^+^ NPC (n=120)** | |  |  |
| --- | --- | --- | --- | --- | --- |
| **Characteristic** | **Total** | **NETs** | | ***χ^2^*** | ***P*-value** |
|  |  | **Low**  **n=86 (71.1%)** | **High**  **n=34(28.9%)** |  |  |
| **Age(y)** |  |  |  | 0.536 | 0.464 |
| <50 | 75 | 52 | 23 |  |  |
| ≥50 | 45 | 34 | 11 |  |  |
| **Gender** |  |  |  | 1.653 | 0.199 |
| female | 27 | 22 | 5 |  |  |
| male | 93 | 64 | 29 |  |  |
| **Clinical Stage** |  |  |  | 0.053 | 0.818 |
| II-III | 58 | 41 | 17 |  |  |
| IV | 62 | 45 | 17 |  |  |
| **T Stage** |  |  |  | 0.198 | 0.656 |
| T1-T2 | 13 | 10 | 3 |  |  |
| T3-T4 | 107 | 76 | 31 |  |  |
| **N Stage** |  |  |  | 0.750 | 0.386 |
| N0-N1 | 56 | 38 | 18 |  |  |
| N2-N3 | 64 | 48 | 16 |  |  |
| **M Stage** |  |  |  | 0.038 | 0.846 |
| M0 | 117 | 84 | 33 |  |  |
| M1 | 3 | 2 | 1 |  |  |
| **EBV DNA**  **(copies/ml)** |  |  |  | 0.230 | 0.632 |
| ≤8471 | 70 | 49 | 21 |  |  |
| >8471 | 50 | 37 | 13 |  |  |

**Abbreviations:** NETs: neutrophil extracellular traps; NPC: nasopharyngeal carcinoma.

**Table S2. Association of NETs expression with clinicopathological features in patients with** **EBVaGC.**

|  |  | **EBVaGC (n=69)** | |  |  |
| --- | --- | --- | --- | --- | --- |
| **Characteristic** | **Total** | **NETs** | | ***χ^2^*** | ***P*-value** |
|  |  | **Low**  **n=35(50.7%)** | **High**  **n=34(49.3%)** |  |  |
| **Age(y)** |  |  |  | 5.280 | **0.022** |
| <60 | 40 | 25 | 15 |  |  |
| ≥60 | 29 | 10 | 19 |  |  |
| **Gender** |  |  |  | 2.552 | 0.110 |
| female | 13 | 4 | 9 |  |  |
| male | 56 | 31 | 25 |  |  |
| **Clinical Stage** |  |  |  | 0.348 | 0.555 |
| I-II | 28 | 13 | 15 |  |  |
| III-IV | 41 | 22 | 19 |  |  |
| **T Stage** |  |  |  | 1.051 | 0.305 |
| T0-T2 | 18 | 11 | 7 |  |  |
| T3-T4 | 51 | 24 | 27 |  |  |
| **N Stage** |  |  |  | 0.123 | 0.726 |
| N0-N1 | 31 | 15 | 16 |  |  |
| N2-N3 | 38 | 20 | 18 |  |  |
| **M Stage** |  |  |  | 0.001 | 0.970 |
| M0 | 63 | 32 | 31 |  |  |
| M1 | 6 | 3 | 3 |  |  |
| **HER-2** |  |  |  | 0.396 | 0.529 |
| low | 40 | 19 | 21 |  |  |
| high | 29 | 16 | 13 |  |  |

**Abbreviations:** NETs: neutrophil extracellular traps; EBVaGC: EBV-associated gastric carcinoma.
